# Cost-effectiveness of mask mandates on subways to prevent SARS-CoV-2 transmission in the United States

**DOI:** 10.1371/journal.pone.0302199

**Published:** 2024-05-15

**Authors:** Joohyun Park, Heesoo Joo, Daniel Kim, Sundari Mase, Deborah Christensen, Brian A. Maskery

**Affiliations:** 1 Division of Global Migration Health, Centers for Disease Control and Prevention, Atlanta, Georgia, United States of America; 2 Oak Ridge Institute for Science and Education, Oak Ridge, Tennessee, United States of America; 3 Georgia Institute of Technology, H. Milton Stewart School of Industrial and Systems Engineering, Atlanta, Georgia, United States of America; Curtin University of Malaysia, MALAYSIA

## Abstract

**Background:**

Community-based mask wearing has been shown to reduce the transmission of SARS-CoV-2. However, few studies have conducted an economic evaluation of mask mandates, specifically in public transportation settings. This study evaluated the cost-effectiveness of implementing mask mandates for subway passengers in the United States by evaluating its potential to reduce COVID-19 transmission during subway travel.

**Materials and methods:**

We assessed the health impacts and costs of subway mask mandates compared to mask recommendations based on the number of infections that would occur during subway travel in the U.S. Using a combined box and Wells-Riley infection model, we estimated monthly infections, hospitalizations, and deaths averted under a mask mandate scenario as compared to a mask recommendation scenario. The analysis included costs of implementing mask mandates and COVID-19 treatment from a limited societal perspective. The cost-effectiveness (net cost per averted death) of mandates was estimated for three different periods based on dominant SARS-CoV-2 variants: Alpha, Beta, and Gamma (November 2020 to February 2021); Delta (July to October 2021); and early Omicron (January to March 2022).

**Results:**

Compared with mask recommendations only, mask mandates were cost-effective across all periods, with costs per averted death less than a threshold of $11.4 million (ranging from cost-saving to $3 million per averted death). Additionally, mask mandates were more cost-effective during the early Omicron period than the other two periods and were cost saving in January 2022. Our findings showed that mandates remained cost-effective when accounting for uncertainties in input parameters (e.g., even if mandates only resulted in small increases in mask usage by subway ridership).

**Conclusions:**

The findings highlight the economic value of mask mandates on subways, particularly during high virus transmissibility periods, during the COVID-19 pandemic. This study may inform stakeholders on mask mandate decisions during future outbreaks of novel viral respiratory diseases.

## Introduction

Following the emergence of COVID-19 in the United States in early 2020, federal, state, and local authorities made efforts to mitigate the transmission of SARS-CoV-2 in order to prevent morbidity and mortality associated with this novel emerging pathogen. These measures included the issuance of guidance and recommendations as well as various mandatory preventive measures, such as stay-at-home orders, gathering limits, and mandates for face masks [1–3]. One widely adopted preventive measure at both the state and federal levels was the implementation of mask mandates. In April 2020, the U.S. Centers for Disease Control and Prevention (CDC) recommended the use of face masks in public settings [4]. Subsequently, many states and counties issued their own requirements for individuals to wear masks. In total, 38 states and the District of Columbia had implemented mandates with specifics of enforcement for public mask wearing by the end of 2020 [5]. CDC issued an order requiring the wearing of masks by individuals on public transportation conveyances traveling into, within, or out of the United States, and at transportation hubs nationwide, which was in effect between February 2021 and April 2022 [6,7].

Several studies have investigated the effectiveness of public mask wearing in reducing the transmission of COVID-19. Research conducted in various settings and countries has consistently demonstrated that widespread mask wearing in public can contribute to reducing the spread of SARS-CoV-2, leading to a decrease in incident cases, hospitalizations, and deaths [8–13]. However, despite several economic evaluations of nonpharmaceutical interventions for COVID-19, only a limited number of studies included public mask wearing as a component of their research [14,15]. These studies have primarily examined broader community contexts [16], college campuses [17], or other countries [18–20]. We found no studies that specifically investigated the cost-effectiveness of mask mandates in the context of public transportation conveyances. The CDC transportation-specific Mask Order included all conveyances, including aircraft, buses, trains, and subways. We investigated the cost-effectiveness of mask mandates in subways as a starting point. Subways, like all public transportation conveyances, play a significant role in people’s daily lives, especially in large cities. Traveling on multi-person conveyances increases a person’s risk of being infected with and spreading COVID-19 by bringing persons in close contact with others. This may occur for prolonged periods during which they may not be able to distance themselves from individuals seated nearby [6]. Addressing this knowledge gap may help promote evidence-based decision-making in the future to protect the public’s health.

We evaluated the cost-effectiveness of implementing a mask mandate by examining the potential reduction of COVID-19 transmission during subway travel. By examining the potential benefits and costs, we aimed to provide insights into the value of implementing mask mandates on subways during the COVID-19 pandemic. Additionally, we examined how the cost-effectiveness of a mask mandate varied under different circumstances by considering changes in ridership patterns, SARS-CoV-2 infection prevalence, population immunity, mask usage, and in the predominant strain of the SARS-CoV-2 virus.

## Materials and methods

We considered two scenarios in the United States: first, the “Mask mandate”, where mask-wearing by subway riders is required, and second, the “Mask recommendation”, where mask-wearing by subway riders is optional but recommended. We estimated the health impacts and costs of the two scenarios from a partial societal perspective and calculated the incremental cost-effectiveness ratio (ICER). We examined monthly estimates and explored the effects of mask-wearing over a time horizon of 11 non-continuous months corresponding to when different strains of the SARS-CoV-2 virus were dominant: first, during the period with Alpha, Beta, and Gamma variants (November 2020 to February 2021); followed by the Delta variant (July to October 2021); and finally, the early Omicron subvariants (January to March 2022).

To evaluate the health impacts of the intervention of mandated mask wearing in subways, we estimated the monthly numbers of COVID-19 cases, hospitalizations, and deaths for each scenario. Initially, we estimated, by month, the probability of SARS-CoV-2 infection for passengers in a subway car assuming the presence of one infectious person, using an airborne SARS-CoV-2 transmission model developed by Peng et al. [21,22]. This model combines a box model for estimating indoor viral aerosol concentration with the Wells-Riley infection model [21]. The authors provided an online spreadsheet model that can be applied to different settings. We adapted the model to simulate different periods during the COVID-19 pandemic, including variation in the dominant variant, population immunity, conveyance occupancies, and the fraction of people wearing masks in public without the mandate (i.e., recommendation only). We assumed that 95% of subway ridership would wear masks with a mandate across the periods and conducted extensive sensitivity analyses around this parameter. Our model input parameters are summarized in Table 1 and further detailed in S1 File in S1 Data.

**Table 1:** Parameter values and sources used to estimate cost-effectiveness of mask mandates on subways.

Parameters	Unit	Baseline	LB	UB	Distribution	Source
**Parameters used for estimating COVID-19 infections, hospitalizations, and deaths** ^a^						
Subway car dimension						
Length	meter	18.4	14.6	22.9	beta-PERT	[23–27]
Width	meter	2.8	2.6	3.1	beta-PERT	
Height	meter	3.3	2.1	3.7	beta-PERT	
Duration of travel	min	24.4	18.3	30.5	beta-PERT	[28], assumption
Ventilation with outside air	per hour	18	11	28	beta-PERT	[29–31]
Number of passengers per subway car ^b^	people	22‒59	17‒55	28‒74	beta-PERT	[32–36], assumption
Fraction of population immune (%) ^c^						
Nov 2020-Feb 2021	%	14.7	11.1	19.8	beta-PERT	[37]
Jul-Oct 2021	%	51.0	47	56	beta-PERT	[38]
Jan-Mar 2022	%	21.8	20.7	23.4	beta-PERT	[37]
SARS-CoV-2 exhalation rate by an infectious person resting and breathing	quanta/ hour	18.6	8.4	48.1	beta-PERT	[21]
Quanta enhancement due to variants ^b^						[21,39–41]
Nov 2020-Feb 2021	-	1.0	1.0	1.0	Constant	
Jul-Oct 2021	-	2.0	2.0	2.0	Constant	
Jan-Mar 2022	-	2.5	2.5	2.5	Constant	
Exhalation mask efficiency ^d^	%	50	37.5	62.5	beta-PERT	[21,42,43], assumption
Inhalation mask efficiency ^e^	%	30	22.5	37.5	beta-PERT	[21,42,43], assumption
People wearing masks in subway						
with mask mandate	%	95	90	100	beta-PERT	Assumption
without mask mandate ^b^	%	32‒62	16‒34	39‒85	beta-PERT	[44–46]
Probability of COVID-19 infection ^b^^,^ ^f^	%	0.1‒1.9	0.1‒1.5	0.1‒2.6	N/A (Calculated)	Calculated
Hospitalization rate given infection ^b^^,^ ^g^	%	1.7‒6.1	0.4‒4.5	0.6‒7.6	N/A (Calculated)	Calculated
Infection fatality ratio ^b^^,^ ^h^	%	0.14‒0.78	0.11‒0.58	0.18‒0.97	beta-PERT	[47–51], assumption
**Cost parameters**						
Reusable face mask (per count) ^i^	$	4.0	0.6	16.7	Gamma	[52]
Disposable face mask (per count)	$	0.3	0.1	2.0	Gamma	[52,53]
People wearing reusable masks	%	34	17	58	beta-PERT	[54]
Communication/signage ^j^	$	1,856,273	-	-	N/A (calculated)	[55–58]
Fraction experiencing disutility from wearing masks	%	14	10	17	beta-PERT	[59], assumption
WTP to ride on a subway car without a mask mandate for individuals with disutility	$	1.5	0.5	27.5	Gamma	[59–61]
Cost of COVID-19 treatment						
Outpatient	$	1,008	927	3,045	Gamma	[62–64]
Hospitalized	$	24,826	19,934	41,611	Gamma	[65–67]
Death	$	27,017	25,527	32,015	Gamma	[65,68]

^as^ We used the airborne COVID-19 transmission model developed by Peng et al. [21], which is accessible as an online spreadsheet model. Details of the model are described in previous studies [21,22].

^b^ Some parameters changed over time, such as month or period. The parameters specified by month or period are described in S1 File in S1 Data.

^c^ Population immunity was estimated for each study period based on published estimates of the percentages of the U.S. population with effective protection against SARS-CoV-2 infection over time, considering vaccine- and infection-acquired immunity, as well as waning immunity.

^d^ Mask efficiencies in reducing virus emission from nose and mouth of an infectious person.

^e^ Mask efficiencies in reducing virus inhalation by a potentially exposed person for virus in aerosol particles floating in the air.

^f^ The incidence of COVID-19 infections in the United States was estimated by back-calculating from reported deaths and period-specific infection fatality ratios assuming an average three-week lag between infection and death. Details of the estimation are described in S1 File in S1 Data.

^g^ The hospitalization rate given infection was estimated by dividing the reported number of new hospital admissions by the estimated number of COVID-19 infections one week prior, assuming a lag of about one week between infection and hospitalization. Details of the estimation are described in S1 File in S1 Data.

^h^ Infection fatality ratios (IFR) were based on published literature. For the months where the IFR information was not available from the literature, we used linear interpolation to estimate the monthly IFR. Details of the estimation are described in S1 File in S1 Data.

^i^ Reusable masks were assumed to be used for a month.

^j^ The cost of communication or signage was calculated by multiplying the price for printing posters by the total number of posters in subways; the range was determined by component price estimates. A total cost of $1,856,273 was applied to the first month of the analysis (November 2020). We then assumed that25% of this amount would be incurred for each subsequent month as maintenance costs. Details of the estimation are described in S3 File in S1 Data.

LB, lower bound; UB, upper bound; PERT, program evaluation and review technique; min, minute; WTP, willingness-to-pay.

Due to lack of specific data for U.S. subway passengers, we applied the national average COVID-19 prevalence in the United States to the monthly numbers of subway passengers to estimate the number of infectious subway passengers by month. We estimated the number of SARS-CoV-2 infections in the United States by back-calculating the number of infections from reported deaths using estimates of the COVID-19 infection fatality ratio (IFR) that varied across different periods (Table 1 and S1 File in S1 Data) [47–51]. We assumed an average of three weeks between infection and death [69–71]. Further details are described in S1 File in S1 Data.

Using the estimated probability of SARS-CoV-2 infection given exposure to an infectious individual in a subway car, we estimated the monthly numbers of SARS-CoV-2 infections for U.S. subways under two scenarios, “Mask mandate” and “Mask recommendation”. The estimates of new infections were based on the monthly numbers of subway cars carrying an infectious person in the United States, passengers who rode on a subway car with an infectious person, and non-immune and potential exposed passengers. The fraction of population with immunity was based on previously reported studies [37,38]. The detailed estimation process is described in S2 File in S1 Data.

We estimated the number of subway cars that would have an infectious person during each month by comparing the national average prevalence estimate to the average number of passengers per subway car. To simplify the analysis, we assumed that if a subway car had an infectious person, it would have exactly one infectious individual. This assumption was relaxed in a sensitivity analysis. The estimated SARS-CoV-2 infections that would occur during subway travel were further subdivided into hospitalized cases, fatal cases, and non-hospitalized cases (outpatient visits only). The number of non-medically attended patients were quantified, but their costs were not considered, given the relatively lower costs of obtaining over-the-counter drugs to alleviate COVID-19 related symptoms and because some would be asymptomatic.

The costs of implementing mask mandates on subways included face masks, communication or signage in subway cars (e.g., posters or interior car cards), and passenger disutility from wearing masks (i.e., opportunity costs for those who would prefer not to wear masks). We estimated monthly incremental costs of reusable and disposable masks among U.S. subway passengers under a mask mandate scenario, using the National Transit Database (NTD) monthly heavy rail (i.e., subway) ridership data [72], U.S. market prices, and the proportions of individuals who would not wear a mask without the mandate but would wear a mask with the mandate [52,53]. The costs associated with communication or signage in subways were based on U.S. market prices or information available in published literature [55–58]. The opportunity cost was extrapolated from a previous survey study that examined respondents’ willingness-to-pay (WTP) more for a flight ticket in which masking would be optional to a hypothetical alternative flight in which masking would be required [59–61]. Further details are provided in S3 File in S1 Data.

We also estimated the costs of COVID-19 treatment in each scenario, “Mask mandate” and “Mask recommendation”. The treatment costs were estimated by multiplying the estimated number of COVID-19 outpatient cases, hospitalizations, and deaths by the average costs per patient for COVID-19 treatment, as sourced from published literature [62–67,73]. S3 File in S1 Data provides further details on the cost estimation.

For the economic evaluation, we compared the health outcomes (including the number of COVID-19 infections, hospitalizations, and deaths) with the total costs (including the intervention costs and COVID-19 treatment costs) between the two scenarios (“Mask mandate” vs. “Mask recommendation”). We calculated ICER estimates, expressed as the net cost per averted death. We adopted the central and lower bound values of statistical life (VSL, $11.4 million and $5.3 million in 2020, respectively) as the WTP thresholds for identifying cost-effective interventions [74]. Therefore, if the ICER was below $11.4 million, “Mask mandate” was considered cost-effective (or $5.3 million using the lower bound threshold). Alternatively, if there were net cost savings (i.e., negative incremental total costs and positive incremental health impacts), “Mask mandate” was considered as a dominant, or cost-saving. strategy. Our analysis was conducted from a limited societal perspective, considering non-medical costs of intervention and direct medical costs associated with treating COVID-19. We reported costs in 2020 U.S. dollars.

We developed the model using Microsoft Excel and R software. The R software was used to conduct one-way deterministic sensitivity analysis based on the lower and upper bound estimates provided in S1 and S3 Files in S1 Data. In addition, we conducted multivariate probabilistic sensitivity analyses to consider the combined effect of simultaneously varying input parameters in the model. We performed 5,000 iterations using @RISK software (version 7), an add-in tool for Microsoft Excel.

We identified some key assumptions that could affect our model for additional sensitivity analyses. First, we varied our assumption that exactly one passenger would be infectious on each subway car, and assumed that exactly two, three, four, or more passengers were infectious on each car. Given the low prevalence of infectious individuals at any one time relative to the number of subway car occupants, we believe that the single infectious passenger example would be the most common scenario. We maintained the same number of total infectious individuals per month as in our baseline estimates by adjusting the number of subway cars with infectious individuals in this sensitivity analysis (e.g., if we assumed two infectious passengers per subway car, the number of subway cars with an infectious passenger would be reduced by 50% to maintain a consistent number of total monthly infectious passengers corresponding to the national average prevalence rate).

Furthermore, given the possibility of a decrease in mask-wearing over time even with a mandate, we conducted additional sensitivity analysis to address this possibility. Our base assumption was that 95% would wear masks (ranging from 90% to 100%). We explored a scenario where only half of those who would not choose to wear masks if recommended would comply with the mask mandate. The associated compliance rate estimates were 81% in the Alpha, Beta, and Gamma period (vs. 62% without a mandate), 64% in the Delta period (vs. 29%), and 66% in the early Omicron period (vs. 32%). For this analysis, we also assumed lower disutility costs (50% of the base case) since those most opposed to wearing masks would be less likely to comply. This approach adds context to potential ICERs of mask mandates if mask wearing rates are lower than expected and proportional to “Mask recommendation” rates. Further details are in S4 File in S1 Data. We also estimated the cost-effectiveness of a mask mandate scenario in comparison to no intervention, assuming no one would wear masks without a mandate.

Lastly, we considered estimates of IFR, deaths, and hospitalizations associated with COVID-19 specific to metropolitan areas (as opposed to the national estimates used in the main analysis). This approach allowed us to examine the potential limitation of using national average estimates of COVID-19 prevalence, hospitalizations, and deaths compared to using estimates tied to metropolitan areas, which may be more consistent with large cities with subway systems. Details of the estimation are described in S5 File in S1 Data.

## Results

Table 2 presents the estimated health outcomes and costs associated with each scenario ("Mask mandate" and "Mask recommendation"), as well as the incremental health outcomes, costs, and ICERs of "Mask mandate" versus "Mask recommendation." All estimates were presented on a monthly basis for the three study periods: the Alpha, Beta, and Gamma period (November 2020 to February 2021); the Delta period (July to October 2021); and the early Omicron period (January to March 2022).

**Table 2:** Estimated health outcomes and costs of mask mandates on subways.

	Alpha, Beta, Gamma Period				Delta Period				Early Omicron Period		
	Nov 2020	Dec 2020	Jan 2021	Feb 2021	Jul 2021	Aug 2021	Sep 2021	Oct 2021	Jan 2022	Feb 2022	Mar 2022
Subway ridership (Thousand)*	94,958	96,182	92,004	86,177	153,468	149,195	160,808	184,490	136,336	156,069	186,855
Passengers who rode on a subway with an infectious person (Thousand)*	7,881	10,606	10,000	5,476	6,195	15,883	20,947	18,353	122,672	123,546	72,646
Non-immune passengers (Thousand)*	6,723	9,047	8,530	4,671	3,035	7,783	10,264	8,993	95,929	96,613	56,809
Unique passengers (Thousand)†	1,187	1,202	1,150	1,077	1,918	1,865	2,010	2,306	1,704	1,951	2,336
**Health outcomes**											
“Mask mandate”											
SARS-CoV-2 infections	1,446	1,945	1,834	1,004	1,305	3,346	4,413	3,867	51,559	51,926	30,533
COVID-19 hospitalizations	59	78	67	33	79	200	176	113	850	456	147
COVID-19 deaths	11	15	14	8	9	24	31	27	148	78	43
“Mask recommendation”											
SARS-CoV-2 infections	2,168	2,917	2,750	1,506	2,726	6,989	9,218	8,077	104,368	105,112	61,807
COVID-19 hospitalizations	88	116	101	50	165	418	368	235	1,721	922	297
COVID-19 deaths	17	22	21	11	20	50	65	57	300	158	87
**Costs (Thousand USD)**											
** Intervention cost**											
Purchasing face masks, (a)	$2,839	$2,876	$2,751	$2,577	$9,191	$8,935	$9,631	$11,049	$7,748	$8,869	$10,619
Disutility of wearing masks, (b)	$10,025	$10,154	$9,713	$9,098	$16,202	$15,751	$16,977	$19,477	$14,393	$16,476	$19,726
Posters in subway cars, (c)	$1,856	$464	$464	$464	$464	$464	$464	$464	$464	$464	$464
** COVID-19 treatment cost**											
“Mask mandate”, (d)	$2,428	$3,320	$3,010	$1,444	$2,683	$7,402	$7,566	$5,015	$51,790	$22,801	$6,987
“Mask recommendation”, (e)	$3,641	$4,978	$4,513	$2,165	$5,604	$15,460	$15,802	$10,474	$104,836	$46,154	$14,143
** Total cost**											
“Mask mandate”, (f) = (a)+(b)+(c)+(d)	$17,149	$16,814	$15,938	$13,582	$28,540	$32,552	$34,638	$36,005	$74,395	$48,610	$37,796
“Mask recommendation”, (g) = (e)	$3,641	$4,978	$4,513	$2,165	$5,604	$15,460	$15,802	$10,474	$104,836	$46,154	$14,143
**Outcomes relative to "Mask recommendation"**											
** Health outcomes**											
SARS-CoV-2 infections averted	722	972	916	502	1,421	3,643	4,804	4,210	52,809	53,186	31,274
COVID-19 hospitalizations averted	29	39	34	17	86	218	192	123	871	467	150
COVID-19 deaths averted, (h)	6	7	7	4	10	26	34	29	152	80	44
** Costs (Thousand USD)**											
Incremental cost of intervention	$14,720	$13,494	$12,928	$12,138	$25,857	$25,150	$27,072	$30,990	$22,605	$25,810	$30,809
Incremental cost of treatment	-$1,213	-$1,658	-$1,503	-$721	-$2,921	-$8,058	-$8,236	-$5,459	-$53,046	-$23,354	-$7,156
Incremental total cost, (i)	$13,508	$11,836	$11,425	$11,417	$22,936	$17,092	$18,836	$25,531	-$30,441	$2,456	$23,653
** ICER, (j) = (i)/(h) (Thousand USD/death averted)**	**$2,404**	**$1,591**	**$1,653**	**$3,045**	**$2,249**	**$659**	**$555**	**$866**	**Cost saving**	**$31**	**$536**

* The monthly subway ridership data was obtained from the National Transit Database (NTD). The number of passengers who rode on a subway with an infectious person, as well as the number of non-immune passengers, were estimated using the estimated probabilities of COVID-19 infections in a subway car and the estimated probability of being infectious in the United States. Further details and assumptions are described in S2 File in S1 Data.

† The number of unique passengers per month was estimated by dividing the NTD monthly ridership data by 80 (2×2×20), assuming that, on average, each passenger had one transfer per trip, would complete one round trip each day, and used subways for 20 days per month (corresponding to working days). This assumption was made due to the nature of the NTD ridership data, which counted passengers each time they boarded a vehicle, regardless of the number of vehicles used for their journey from origin to destination (i.e., unlinked passenger trips). Further details are in S3 File in S1 Data.

ICER, incremental cost-effectiveness ratio; USD, U.S. dollar.

### Health outcomes

A monthly estimate of the number of subway passengers non-immune to SARS-CoV-2 infection and who shared a subway car with an infectious passenger (i.e., potentially exposed passengers) varied within and across the different periods and generally increased over time. It ranged from approximately 5 to 9 million passengers in the Alpha, Beta, and Gamma period, 3 to 10 million passengers in the Delta period, and 57 to 97 million passengers during the early Omicron period (Table 2). As expected, the number of COVID-19 infections, hospitalizations, and deaths increased as the number of potentially exposed passengers increased.

In addition, higher numbers of subway-associated infections, hospitalizations, and deaths were estimated under the “Mask recommendation” scenario compared to the “Mask mandate” scenario. Under the “Mask mandate” scenario, the estimated monthly number of persons infected in subways during the Alpha, Beta, and Gamma period ranged from approximately 1,004 to 1,945, which would result in between 33 and 78 passengers requiring hospitalization and between 8 and 15 deaths due to COVID-19. For the Delta period, the number of subway-associated infections ranged from 1,305 to 4,413, hospitalizations ranged from 79 to 200, and deaths ranged from 9 to 31. Furthermore, during the early Omicron period, estimates ranged from 30,533 to 51,926 subway-associated infections, 147 to 850 hospitalizations, and 43 to 148 deaths. Overall, “Mask mandate” was estimated to reduce the number of infections, hospitalizations, and deaths by about 33% during the Alpha, Beta, and Gamma period, by 52% during the Delta period, and by 51% during the early Omicron period. The month in which “Mask mandate” would result in the highest number of infections, hospitalizations, and deaths averted (52,809 infections, 871 hospitalizations, and 152 deaths) was January 2022 (early Omicron period). In contrast, the smallest numbers would have been averted in February 2021 (Alpha, Beta, and Gamma period, 502 infections, 17 hospitalizations, and 4 deaths). Table 2 presents estimates of averted infections, hospitalizations, and deaths for each month and period.

### Costs

During the three variant-specific periods, we estimated that total costs, including mask wearing and illness costs, of the “Mask mandate” scenario ranged between $14 million (February 2021) and $74 million (January 2022) (Table 2). The costs of face masks and the disutility of wearing masks exceeded the costs of COVID-19 treatment during the Alpha, Beta, Gamma to Delta periods; and these costs accounted for more than 75% of the total costs. During the early Omicron period, when prevalence rates were highest, the cost of COVID-19 treatment contributed more to the total cost estimates and accounted for 70% and 47% of the total costs in January and February 2022, respectively.

**Fig 1:**
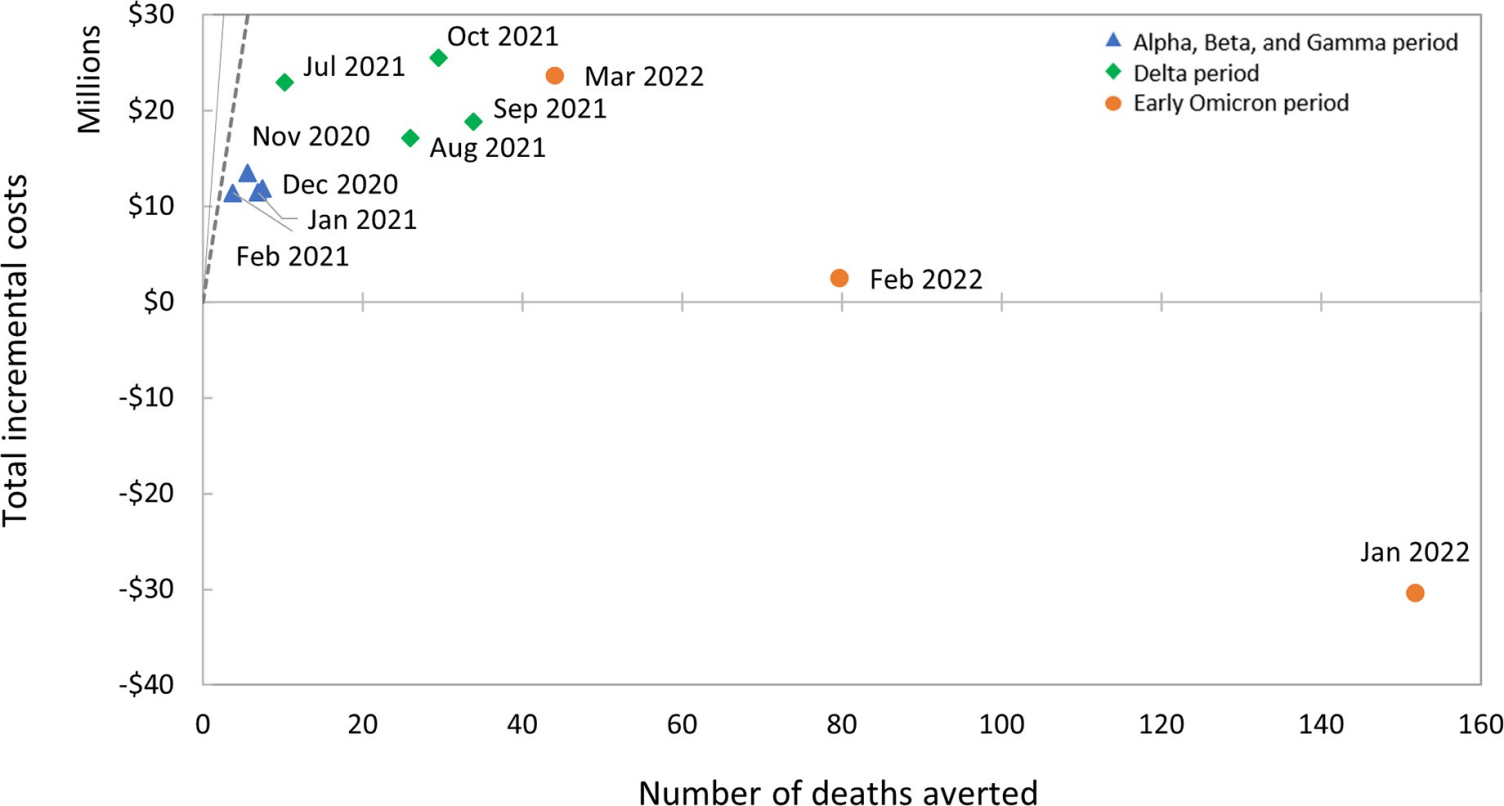
Cost-effectiveness plane by month for mask mandates on subways. The blue triangle represents the Alpha, Beta, and Gamma period, the green diamond represents the Delta period, and the orange circle represents the early Omicron period. The solid gray line represents the central value of the value per statistical life (VSL) at $11.4 million and the dotted gray line indicates the lower value of the VSL at $5.3 million.

The total incremental costs of “Mask mandate” compared to “Mask recommendation” ranged from $11 million to $14 million during the Alpha, Beta, and Gamma period, from $17 million to $26 million during the Delta period, and from cost-saving to $24 million during the early Omicron period (Fig 1 and Table 2).

### Incremental cost-effectiveness ratio

Relative to “Mask recommendation”, the ICER estimate for the mask mandate in subways varied from $1.6 million to $3.0 million per death averted during the Alpha, Beta, and Gamma period and from $0.6 to $2.2 million per death averted during the Delta period. For the early Omicron period, we estimated potential cost savings for January 2022 and ICERs of $30,783 and $535,988 per death averted in February and March 2022, respectively. Each of these ICER estimates were less than our assumed WTP threshold ($11.4 million). Therefore, “Mask mandate” would be considered cost-effective compared to “Mask recommendation” throughout the study periods.

### Sensitivity analyses

**Fig 2:**
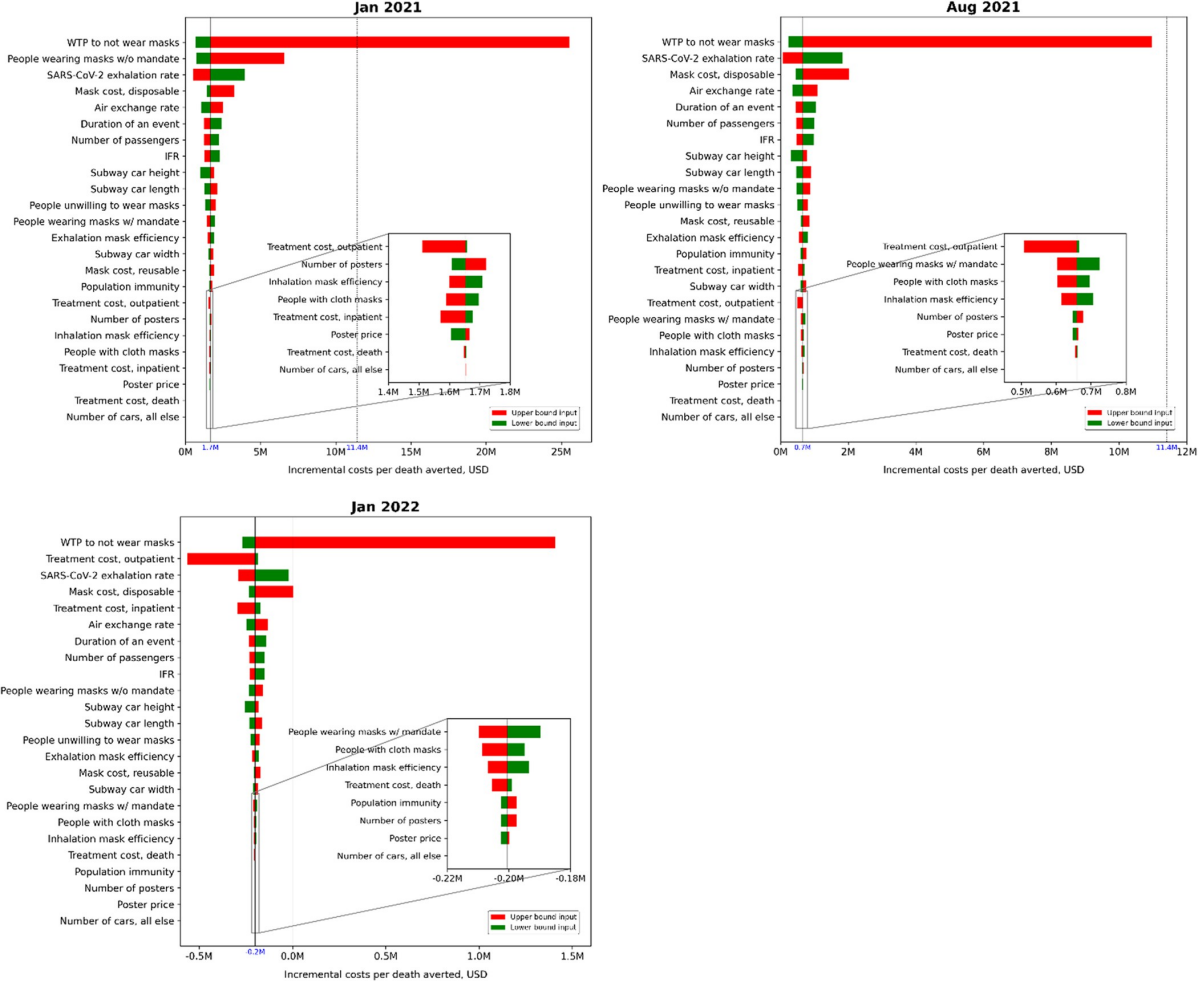
One-way sensitivity analysis results for the cost-effectiveness of “Mask mandate” relative to “Mask recommendation” on subways for selected months in each period. Only results for representative months of the three periods are presented here due to limited space (January 2021 for the Alpha, Beta, and Gamma period, August 2021 for the Delta period, and January 2022 for the early Omicron period); the results for all other months can be found in S1 Fig in S1 Data. The solid lines represent the baseline incremental costs per death averted (i.e., ICER) and the dotted lines indicate the cost-effectiveness threshold (i.e., central value of the value per statistical life, $11.4 million). Each horizontal bar illustrates the changes in the ICER as each input parameter is varied over the uncertainty range of the lower and upper bound estimates, while maintaining all other parameters at their base case values.

The one-way sensitivity results are summarized in tornado diagrams that show that the disutility from mask wearing (based on WTP estimates to switch from riding on a subway with mandatory mask wearing to a subway with optional mask wearing) had the greatest contribution to our ICER estimate uncertainty, regardless of the month (Fig 2 and S1 Fig in S1 Data). The next two most influential input parameters varied by period. In the Alpha, Beta, and Gamma period, the proportion of people wearing masks under “Mask recommendation” and the SARS-CoV-2 exhalation rate (i.e., the rate of viral load emitted to indoor air, quanta per hour) by an infectious person were the next most influential parameters. During the Delta period, the SARS-CoV-2 exhalation rate and the cost of disposable masks were the second and third most influential parameters. In January 2022, during the early Omicron period, outpatient treatment costs and the SARS-CoV-2 exhalation rate ranked as the second and third most influential parameters. In February and March 2022, the SARS-CoV-2 exhalation rate and the cost of disposable masks were identified as the second and third most influential parameters.

**Fig 3:**
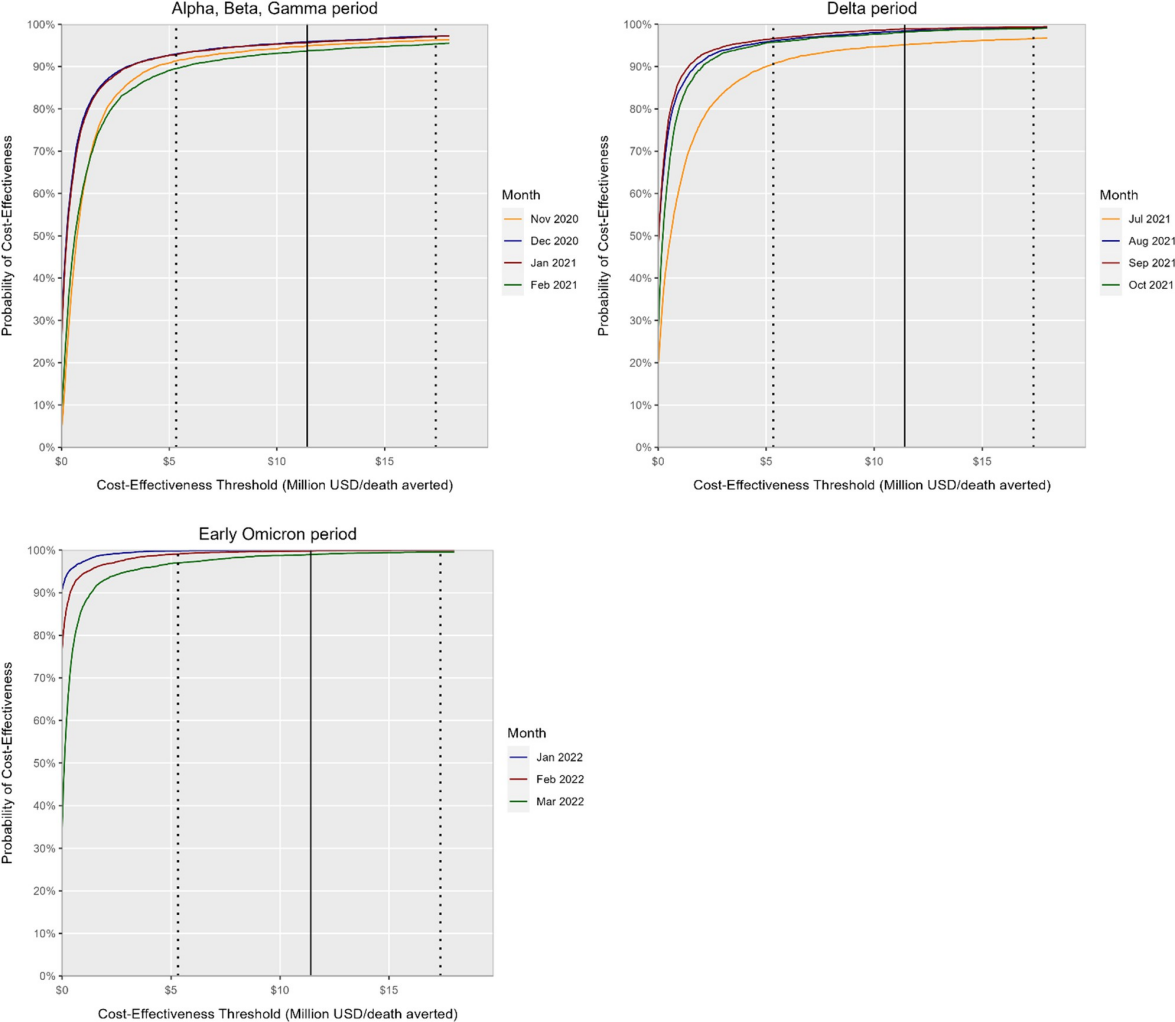
Probabilistic sensitivity analysis results, cost-effectiveness acceptability curves, for the cost-effectiveness of “Mask mandate” relative to “Mask recommendation” on subways. The solid black lines indicate the central value of the value per statistical life (VSL) which was set at $11.4 million in 2020 USD, serving as the threshold for determining the cost-effectiveness of the intervention. The dotted black lines represent the lower VSL estimate (left; $5.3 million) and the higher VSL estimate (right; $17.4 million), respectively. At a threshold of $11.4 million per death averted,”Mask mandate” is cost-effective in 95% of 5,000 iterations for Nov 2020, 96% for Dec 2020, 96% for Jan 2021, 94% for Feb 2021, 95% for July 2021, 98% for Aug 2021, 99% for Sep 2021, 98% for Oct 2021, 99.9% for Jan 2022, 99.8% for Feb 2022, and 99.0% for Mar 2022.

The probabilistic sensitivity analysis (5,000 iterations) show that our results were robust across parameter uncertainty ranges. The cost-effectiveness acceptability curves for each period are shown in Fig 3 and the incremental cost-effectiveness scatter plots are presented in S2 Fig in S1 Data. The figures also show the WTP threshold. For all iterations of ICER estimates below this threshold, “Mask mandate” would be considered cost-effective compared with “Mask recommendation”. More than 90% of ICER estimate iterations across all periods were below this threshold. Specifically, in January and February 2022,–“Mask mandate” was cost-effective in 99% of the iterations.

When we changed the assumption of exactly one infectious passenger on subway cars with potentially exposed passengers to a greater number up to 20% of the base-case number of passengers (i.e., total number of passengers per car), the ICERs estimated for the mask mandate slightly increased, particularly during the Alpha, Beta, and Gamma period (S3 Fig in S1 Data). The highest increase was observed in February 2021, which increased from $3.0 million per averted death with one infectious person per subway car to $3.6 million per averted death with four infectious persons per car. The increasing trends were less noticeable during the Delta and the early Omicron periods. In July 2021, the ICER estimate increased from $2.2 million per averted death with one infectious person per subway car to $2.8 million per averted death with seven infectious persons per car. Similarly, in March 2022, it rose from $0.5 million per averted death with one infectious person per subway car to $0.7 million per averted death with 12 infectious persons per car.

When we used lower proportions of people wearing masks for “Mask mandate”, which were proportional to the rates assumed for passengers who wear masks under “Mask recommendation” during the three periods (81%, 64%, and 66%, respectively), the estimated ICERs remained lower than the HHS-recommended WTP threshold, with cost savings observed in January and February in 2022 (S1 Table in S1 Data). Further, even with coverage rates only slightly above what was assumed for the “Mask recommendation” scenario, the”Mask mandate” scenario remained cost effective (S4 Fig in S1 Data). In addition, when assuming that zero people would wear masks without a mandate, the mask mandate scenario was found to be more cost-effective than scenarios where some individuals would wear masks even without a mandate (S2 Table in S1 Data).

The results using the metropolitan-specific estimates of COVID-19 infections, hospitalizations, and deaths were consistent with the main analysis results and resulted in similar ICER estimates (S3 Table in S1 Data).

## Discussion

We estimated potential costs and health outcomes of implementing mask mandates for subway passengers to prevent SARS-CoV-2 transmission during three different periods, accounting for variation in prevalence, population-based immunity, conveyance occupancies, and mask-wearing compliance over time. Compared with “Mask recommendation”, “Mask mandate” was cost-effective, with an estimated ICER ranging from cost-saving to $3 million per averted death, across all periods. These ICER estimates compare favorably to the HHS‒recommended WTP threshold ($11.4 million). In addition, the “Mask mandate” scenario was most cost-effective in the early Omicron period, with cost-savings observed in January 2022, followed by the Delta period and the Alpha, Beta, and Gamma period. The “Mask mandate” scenario continued to be cost-effective even with modest increases in mask usage with the mandate relative to “Mask recommendation”.

The findings indicate that the “Mask mandate” scenario was most cost-effective during the early Omicron period, which had lower ICER estimates compared to the other two periods. This can be attributed to a higher number of averted COVID-19 deaths and greater cost savings on COVID-19 treatment under the mask mandate during the early Omicron period (22 times and 35 times higher, respectively, in January 2022 than in 2021). There has been evidence that the Omicron variant is associated with a higher transmission rate but lower disease severity, including a reduced likelihood of hospitalizations, decreased need for intense respiratory support, shorter hospital stays, and lower risk of death [75–78]. The early Omicron period was also representative of higher ridership and relatively low coverage of mask wearing without a mandate. In addition, the fraction of passengers assumed to be immune dropped considerably relative to the previous Delta period, possibly due to the more transmissible or immune-evading Omicron variant and waning of immunity [37,38].

To demonstrate the potential impact of the mandatory use of masks for subway passengers (i.e., the expectation that all passengers should follow the rule), this study assumed that the majority of subway passengers (95%) would comply with the mandate. We do not have complete data on compliance with mask mandates and compliance rates remain uncertain. The willingness to continue to wear masks, even with a mandate, may decrease over time or during periods with low disease prevalence. The only data we found indicated relatively high rates of mask compliance in New York City subways even late in the outbreak; the authors reported compliance rates of 95% in January 2022 and a weighted average of 90% for the three-month period from January through March 2022 [79]. These periods coincide with a state-level mask mandate in public areas (effective between April 2020 and September 2022) [80,81], as well as the transportation-specific federal mask mandate (effective between February 2021 and April 2022) [6,7]. We also analyzed the impact of lower compliance rates. This is important because jurisdictions may have limited resources to enforce a mask mandate on subways. We found that mask mandates would remain cost-effective even with small increases in compliance rates relative to mask recommendations.

Because many individuals with SARS-CoV-2 infections may not seek treatment, we estimated the incidence of new infections and hospitalization rates rather than relying on reported estimates. Our estimates are in line with literature estimates for the Delta and Omicron periods in that the estimated fraction of population infectious with COVID-19 was significantly higher while the hospitalization rate was lower in the early Omicron period (e.g., 1.9% of the U.S. population was infectious with a 1.7% hospitalization rate for infected individuals in January 2022 compared to 0.3% and 6.0% in August 2021 during the period in which Delta was the predominant variant; S1 File in S1 Data). Because of the larger number of SARS-CoV-2 infections during the early Omicron period, a mask mandate would avert more COVID-19 infections, hospitalizations, and deaths, as well as the concomitant costs of COVID-19 treatment despite the lower hospitalization rates and infection fatality ratios for this variant.

To estimate the number of SARS-CoV-2 infections, we adapted the Peng et al. model to estimate the probability of new infections during subway trips in the United States. The Peng et al. model focused solely on the shared-room airborne transmission of SARS-CoV-2, which occurs through aerosols carrying SARS-CoV-2 originating from the nose and mouth of an infectious individual [21,22]. As SARS-CoV-2 can also be transmitted through close contact (e.g., less than 6 feet) or indirectly through contact with contaminated surfaces (e.g., skin-to-skin contact, or fomites via contact with handrails or seat rests) [82,83], our estimates of SARS-CoV-2 infections may underestimate the extent of infections resulting from these transmission routes. However, transmission through the air encompasses the primary exposures to the virus, including the inhalation of air carrying respiratory droplets and aerosol particles, as well as the deposition of viral particles [34]. Therefore, we believe that our estimates would account for the majority of potential COVID-19 cases in the subway scenario.

Our findings align with previous studies conducted in different contexts, settings, and countries, providing further evidence supporting the use of face masks in public settings to prevent SARS-CoV-2 transmission from a cost-effectiveness perspective [16–20]. Notably, two computational modeling studies conducted in the United States have contributed valuable insights. One focused on community-level transmission across the entire U.S. population, considering varying levels of vaccination coverage [16]. The other examined the cost-effectiveness of various prevention strategies, including mandatory mask wearing on a college campus [17]. Both studies presented strong evidence supporting the cost-effectiveness of mask mandates. For example, they indicated that maintaining face mask use was cost-effective or cost-saving, even after achieving the target vaccination coverage of 70–90% for the entire population [16].

Our study contributes additional evidence by focusing on the public transportation setting, specifically subways. Considering the crucial role of public transportation in people’s daily lives and its potential as a pathway for SARS-CoV-2 transmission, particularly in densely populated major U.S. cities, this analysis may be used to inform policy decisions during future outbreaks and pandemics. One unique aspect of this analysis is the comparator to the mask mandate, which used available data on the fraction of the population who would wear masks in the absence of a requirement for the “Mask recommendation” alternative. In comparison, other studies used a comparator with zero mask wearing, which may not be representative of the decision facing policy makers. A mask recommendation without the mandate would be sufficient to ensure some fraction of the population would wear masks and avoid the disutility costs for individuals who strongly oppose wearing masks. Our findings underscore the significance of face mask usage as a cost-effective strategy to mitigate SARS-CoV-2 transmission on subways, especially in densely populated areas.

This study was subject to several limitations. The study results relied heavily on estimated parameters from multiple sources in the literature or required assumptions by the authors. We used a conservative approach in estimating the base-case value of the parameters. For example, as shown in the one-way sensitivity analysis results, the cost-effectiveness of the mask mandate (i.e., the ICER estimates) is sensitive to the estimated WTP amount for the fraction of the population opposed to wearing face masks and to the proportion of people who would wear masks without the mandate, particularly during the Alpha, Beta, and Gamma period. Given the parameter uncertainties, we considered wide ranges to assess the robustness of the study results and conducted probabilistic sensitivity analyses, which showed that the “Mask mandate” scenario is cost-effective in more than 90% of iterations compared to the HHS‒recommended WTP threshold. Furthermore, the results of our extended sensitivity analyses showed that the “Mask mandate” scenario remained cost-effective after adjusting assumptions such as the number of infectious people in a subway car and greatly reducing the proportion of passengers wearing masks under a mask mandate.

Another limitation was that we did not account for the potential disutility associated with experiencing COVID-19 illness, including physical discomfort, pain, or health consequences after the infection (e.g., post-exertional malaise or cognitive impairment) that could impact individuals’ wellness and quality of life [84]. Furthermore, we did not incorporate non-medical costs, including patient time seeking medical care, transportation expenses, unpaid caregiver time, or potential productivity loss due to illness or caregiving responsibilities [85]. These omissions underestimate the economic and societal benefits of implementing a mask mandate, implying that our ICER estimates are conservative.

While we accounted for the disutility associated with wearing masks, we did not fully consider the potential utility or positive impact of wearing masks for individuals who would prefer to ride on conveyances in which more people are wearing masks. Such benefits were previously estimated based on WTP to fly in an aircraft in which masking was required. The fraction of the surveyed population willing to pay extra to fly on an aircraft with a mandate was more than twice the fraction who reported that they would pay extra to fly on an aircraft where masking was not required [59]. Thus, the disutility to the population opposed to mask wearing may be offset by the utility to the population who would prefer to travel with a mandate in place. However, we did not account for these benefits in this analysis to be conservative.

Lastly, the findings should be interpreted with caution due to the focus on estimating the potential risks of SARS-CoV-2 infection during subway travel. Due to a lack of specific SARS-CoV-2 infection prevalence or health outcomes data for this population, we assumed that more generalized data for the U.S. population would apply to the subway passenger population. However, there may be differences in age, population immunity, or other important characteristics unique to subway passengers. Further, at the outset of the COVID-19 pandemic, when the infection rate was low, the subway “Mask mandate” scenario might have been less cost-effective compared to periods with higher prevalence rates when considering subway transmission apart from other factors. However, additional precautions could still help prevent community spread; thus, even a marginally smaller number of infections during subway travel could contribute to preventing wider spread within the community. Conversely, during periods of high infection (e.g., the early Omicron period evaluated in this analysis), the subway mask mandate could delay, rather than prevent, infections due to ongoing community exposures. Additionally, mask wearing could also help prevent other viral respiratory diseases including influenza [86], such that the benefit of mask wearing might be underestimated in this study.

While this study provides valuable insights into the potential impact on SARS-CoV-2 transmission during subway travel, individuals may also be exposed to the virus in other settings associated with subway usage, such as subway platforms, transport hubs for transferring to buses or trains, or other public transportation conveyances when passengers make transfers. These additional points of exposure before or after subway travel could potentially contribute to a higher risk of infection than our estimates. In this regard, this study may have underestimated the extent of the overall risk associated with subway-related SARS-CoV-2 transmission. Further research might be needed to comprehensively assess the complete spectrum of SARS-CoV-2 transmission risks in various public transportation scenarios.

## Conclusions

This study aims to evaluate the cost-effectiveness of implementing mask mandates on subways during the COVID-19 pandemic. We explored how ICERs varied across different circumstances, including periods when different variants were predominant. Our findings demonstrate substantial value in implementing mask mandates on subways during the COVID-19 pandemic, particularly when the transmissibility of the virus was high, as seen with the emergence of the Omicron variant. By examining both the potential benefits and costs of implementing mask mandates in subways, this research may help stakeholders make informed decisions regarding the implementation of mask mandates during future outbreaks or pandemics of viral respiratory diseases. Furthermore, it may provide valuable insights for future decision making. Future studies may be warranted to explore the cost-effectiveness of mask mandates in different public transportation settings, such as buses, trains, or airplanes. Additional studies could also assess economic impact, feasibility, enforcement costs, potential healthcare savings, societal factors affecting compliance or adaptability, and long-term benefits. This may provide a comprehensive understanding of implementing mask mandates to prevent SARS-CoV-2 transmission during travel on public transportation conveyances.

## Supporting information

S1 Data S1-S5 Files, S1-S4 Fig, S1-S3 Tables. (DOCX)
