# Supplementary material for: Cost-effectiveness of mask mandates on subways to prevent SARS-CoV-2 transmission in the United States
Source: PLoS One. 2024 May 15;19(5):e0302199. doi: 10.1371/journal.pone.0302199 (PMC11095714; doi:10.1371/journal.pone.0302199)
Supplement: S1 Data — (DOCX) [file pone.0302199.s001.docx]

**S1. Parameters used for estimating the probability of COVID-19 infections, hospitalizations, and deaths in subway cars, using the airborne COVID-19 transmission model by Peng et al. [1]**

**Table. Parameter values and sources**

| **Parameters** | **Unit** | **Baseline** | **LB** | **UB** | **Distribution** | **Reference** |
| --- | --- | --- | --- | --- | --- | --- |
| **Environmental parameters** | | | | | | |
| Subway car dimensions |  |  |  |  |  |  |
| Length | meter | 18.4 | 14.6 | 22.9 | beta-PERT | [2-6] |
| Width | meter | 2.8 | 2.6 | 3.1 | beta-PERT |  |
| Height | meter | 3.3 | 2.1 | 3.7 | beta-PERT |  |
| Duration of travel | min | 24.4 | 18.3 | 30.5 | beta-PERT | [7], assumption |
| Ventilation with outside air | per hour | 18 | 11 | 28 | beta-PERT | [8-10] |
| **Parameters related to people and activity in a subway car** | | | | | | |
| Number of passengers per subway car |  |  |  |  |  |  |
| Nov 2020-Feb 2021 | people | 22 | 17 | 28 | beta-PERT | [11, 12], assumption |
| Jul-Oct 2021 | people | 36 | 27 | 45 | beta-PERT | [13], assumption |
| Jan 2022 | people | 48 | 36 | 60 | beta-PERT | [14, 15], assumption |
| Feb 2022 | people | 57 | 43 | 71 | beta-PERT | [14, 15], assumption |
| Mar 2022 | people | 59 | 44 | 74 | beta-PERT | [14, 15], assumption |
| Fraction of population immune to SARS-CoV-2 infection (%) |  |  |  |  |  |  |
| Nov 2020‒Feb 2021 | % | 14.7 | 11.1 | 19.8 | beta-PERT | [16] |
| Jul-Oct 2021 | % | 51.0 | 47.0 | 56.0 | beta-PERT | [17] |
| Jan-Mar 2022 | % | 21.8 | 20.7 | 23.4 | beta-PERT | [16] |
| SARS-CoV-2 exhalation rate by an infectious person resting and breathing | quanta/hour | 18.6 | 8.4 | 48.1 | beta-PERT | [1] |
| Quanta enhancement due to variants |  |  |  |  |  |  |
| Nov 2020‒Feb 2021 | dimensionless | 1.0 | 1.0 | 1.0 | Constant | [1, 18] |
| Jul-Oct 2021 |  | 2.0 | 2.0 | 2.0 | Constant | [1, 18, 19] |
| Jan-Mar 2022 |  | 2.5 | 2.5 | 2.5 | Constant | [1, 20] |
| Exhalation mask efficiency ^b^ | % | 50.0 | 37.5 | 62.5 | beta-PERT | [1, 21, 22], assumption |
| Inhalation mask efficiency ^c^ | % | 30.0 | 22.5 | 37.5 | beta-PERT | [1, 21, 22], assumption |
| Proportion of people wearing masks |  |  |  |  |  |  |
| “Mask mandate” | % | 95.0 | 90.0 | 100 | beta-PERT | Assumption |
| “Mask recommendation” |  |  |  |  |  |  |
| Nov 2020-Feb 2021 | % | 61.8 | 33.8 | 85.2 | beta-PERT | [23, 24] |
| Jul-Oct 2021 | % | 28.5 | 15.6 | 39.3 | beta-PERT | [23-25] |
| Jan-Mar 2022 | % | 31.9 | 17.4 | 44.0 | beta-PERT | [23-25] |
| **Parameters related to COVID-19 disease** | | | | | | |
| Infection fatality ratio |  |  |  |  |  |  |
| Nov 2020 | % | 0.78 | 0.58 | 0.97 | beta-PERT | [26, 27], assumption |
| Dec 2020 | % | 0.77 | 0.57 | 0.96 | beta-PERT | [26, 27], assumption |
| Jan 2021 | % | 0.75 | 0.57 | 0.94 | beta-PERT | [26, 27], assumption |
| Feb 2021 | % | 0.75 | 0.56 | 0.94 | beta-PERT | [26, 28], assumption |
| Jul 2021 | % | 0.72 | 0.54 | 0.90 | beta-PERT | [26, 28], assumption |
| Aug 2021 | % | 0.71 | 0.53 | 0.89 | beta-PERT | [26, 28], assumption |
| Sep 2021 | % | 0.71 | 0.53 | 0.88 | beta-PERT | [26, 28], assumption |
| Oct 2021 | % | 0.70 | 0.53 | 0.88 | beta-PERT | [26, 28], assumption |
| Jan 2022 | % | 0.29 | 0.22 | 0.36 | beta-PERT | [28], assumption |
| Feb 2022 | % | 0.15 | 0.11 | 0.19 | beta-PERT | [28], assumption |
| Mar 2022 | % | 0.14 | 0.11 | 0.18 | beta-PERT | [27, 29, 30], assumption |
| Probability of being infectious |  |  |  |  |  |  |
| Nov 2020 | % | 0.40 | 0.32 | 0.53 |  | Calculated using infection fatality ratio and number of deaths [31] among U.S. population [24] |
| Dec 2020 | % | 0.53 | 0.42 | 0.70 |  |  |
| Jan 2021 | % | 0.52 | 0.41 | 0.69 |  |  |
| Feb 2021 | % | 0.30 | 0.24 | 0.40 |  |  |
| Jul 2021 | % | 0.12 | 0.09 | 0.15 |  |  |
| Aug 2021 | % | 0.30 | 0.24 | 0.41 |  |  |
| Sep 2021 | % | 0.37 | 0.30 | 0.50 |  |  |
| Oct 2021 | % | 0.28 | 0.23 | 0.38 |  |  |
| Jan 2022 | % | 1.91 | 1.53 | 2.55 |  |  |
| Feb 2022 | % | 1.41 | 1.13 | 1.88 |  |  |
| Mar 2022 | % | 0.67 | 0.54 | 0.89 |  |  |
| Hospitalization rate |  |  |  |  |  |  |
| Nov 2020 | % | 4.06 | 3.05 | 5.08 |  | Calculated using estimated infections and new COVID-19 hospital admissions [31] |
| Dec 2020 | % | 3.99 | 2.99 | 4.99 |  |  |
| Jan 2021 | % | 3.66 | 2.75 | 4.58 |  |  |
| Feb 2021 | % | 3.32 | 2.49 | 4.16 |  |  |
| Jul 2021 | % | 6.05 | 4.54 | 7.56 |  |  |
| Aug 2021 | % | 5.98 | 4.48 | 7.47 |  |  |
| Sep 2021 | % | 4.00 | 3.00 | 5.00 |  |  |
| Oct 2021 | % | 3.11 | 2.33 | 3.88 |  |  |
| Jan 2022 | % | 1.65 | 1.24 | 2.06 |  |  |
| Feb 2022 | % | 0.88 | 0.66 | 1.10 |  |  |
| Mar 2022 | % | 0.48 | 0.36 | 0.60 |  |  |

^a^ The airborne transmission model for COVID-19 by Peng et al. [1] is accessible as an online spreadsheet model. Details of the model are described in previous studies [1, 32].

^b^ Mask efficiencies in reducing virus emission from nose and mouth of an infectious person.

^c^ Mask efficiencies in reducing virus inhalation by a susceptible person for virus already in aerosol particles floating in the air.

LB, lower bound; UB, upper bound; PERT, program evaluation and review technique; min, minute

The **dimensions of subway cars** were estimated based on information about subway cars in New York City (Metropolitan Transportation Authority, MTA) [5], Washington D.C. (Washington Metropolitan Area Transit Authority, WMATA) [2], Chicago (Chicago Transit Authority, CTA) [6], and Boston (Massachusetts Bay Transportation Authority, MBTA) [3, 4]. These cities accounted for approximately 90% of the total U.S. ridership in 2019 [33]. The baseline estimates were the unweighted average estimates of the four cities, while the lower and upper bound estimates were the minimum and maximum dimensions from the four cities.

To determine the duration of potential exposure to a person with infectious COVID-19 during subway travel (i.e., **duration of travel)**, we utilized the average one-way commute time to a workplace by subway, which was 48.8 minutes according to the 2019 American Community Survey [7]. Assuming a single transfer per trip, the average travel time per subway car was calculated to be 24.4 minutes after dividing 48.8 minutes by two. We assumed the lower and upper bound estimates to be 75% and 125% of the baseline estimates.

Information about the **ventilation rates** in subway cars (i.e., the number of times that air gets replaced with fresh air per hour) was derived from subway cars in New York City, Chicago, and Boston. In general, subway cars are ventilated either by air filtration, open windows, or opening and closing of doors at stations. The air circulates through subway cars and is replaced with fresh air. The baseline estimate for the air refresh rate (18-times per hour) was based on New York City subway cars [8], because they have the largest subway ridership in the United States, accounting for more than 70% [33]. The lower and upper bounds were obtained from the minimum and maximum ranges of ventilation rates observed in Boston’s subway cars (11- to 28-times per hour) [10]. The ventilation rate in Chicago’s subway cars was 15-times per hour [9].

We estimated the **number of passengers** per subway car during each different study period in order to reflect the changes in subway ridership during the COVID-19 pandemic. We delineated the study into three periods based on dominant viral variants observed: first, during the period with Alpha, Beta, and Gamma variants (November 2020 to February 2021); followed by the Delta variant (July to October 2021); and finally, the early Omicron subvariants (January to March 2022). In Chicago, there were policies implemented during the COVID-19 pandemic to limit the number of passengers per subway car. These restrictions included a limit of 22 passengers per car effective in April 2020, an increased limit of 36 passengers effective from May 2021, and a return to full capacity as of June 2021 [11-13]. These capacity limits were used as the baseline estimates during the first and second study periods. During the early Omicron period, as the capacity limit polices in states were lifted, we estimated the number of passengers per subway car by multiplying the average number of passengers per car by the reduction rate of subway ridership during the pandemic. The difference in the number of passengers per subway car between peak and off-peak hours was approximately 50% of the peak hour figures [34]. Therefore, the average number of passengers per car was assumed to be 75% of the maximum capacity per car, 100 passengers, which was calculated by multiplying the average maximum capacity of subways in New York City, Chicago, and Washington D.C (133 passengers per subway car) [2, 5, 15] by 75%. To estimate the reduction rate of subway ridership during the pandemic, we compared the monthly subway ridership in January (48%), February (57%), and March 2022 (59%) to the figures for the same months in 2019 [33]. This assumed that the same number of subway cars would be operated during 2019 and 2022. The lower and upper estimates are assumed to be between 75% and 125% of the baseline estimates.

**Population immunity** was estimated for each study period based on published estimates of the percentages of the U.S. population with effective protection against SARS-CoV-2 infection over time, taking into account waning immunity and increased immune escape for viral variants [16, 35]. The estimated population immunity of 14.7% in January 2021 [16] was used for the Alpha, Beta, and Gamma period. An estimate of 51% for November 2021 [35] was used for the Delta period, and an estimate of 21.8% for December 2021 was used for the early Omicron period [16]. Lower and upper bound estimates were based on the corresponding 95% credible intervals [16, 35].

We used the best-fit value of **SARS-CoV-2 exhalation rate** (18.6 quanta per hour) by an infectious person who was resting and breathing, as reported by Peng et al. [1]. To account for the uncertainties of relative risk and attack rates, they performed Monte Carlo uncertainty propagation to estimate the uncertainty of the exhalation rate [1], which resulted in 8.4 and 48.1 quanta per hour as the 5^th^ and 95^th^ percentiles, respectively. We used these values as the lower and upper bound estimates, respectively.

The **quanta enhancement factors** compared to the original variant was incorporated in the airborne transmission model for the Delta and early Omicron variants [1]. These enhancement factors were multiplied by the SARS-CoV-2 exhalation rate of an infectious individual to reflect relative increases in exhalation rates due to the COVID-19 variants of concern. Some variants of concerns, such as the Delta and early Omicron variants, appear to be more transmissible than the original SARS-CoV-2 virus or variant [18, 20, 36]. The baseline estimates of the quanta enhancement factors for the Delta and early Omicron variants were taken from published literature [1, 18].

The **exhalation mask efficiency** pertains to the ability of a mask to reduce virus emission from the nose and mouth of an infectious person. The **inhalation mask efficiency** relates to the capacity of a mask to decrease virus inhalation in the context of airborne virus particles that are already present in the surrounding aerosol [1]. In our study, we adopted the baseline estimates from previous studies [1, 21] and assumed lower and upper bounds would range from 75% to 125% of the baseline values.

We estimated the **proportion of people who wore masks** in the U.S. population during each of the three study periods, considering the presence or absence of a mask mandate. Under the “Mask mandate” scenario, we assumed 95% (range: 90%‒100%) of individuals wearing masks. In the absence of a mask mandate (i.e., the “Mask recommendation” scenario), we referenced a prior study that reported the average mask usage percentages for states without mask mandates from July to August 2020 [23]. From this study, we calculated the weighted average of mask usage percentage to be 62.8% and used it as the baseline estimate for the first study period (November 2020‒February 2021). The minimum (33.8%) and maximum (85.2%) values of the mask usage percentages from the study were used as the lower and upper bound estimates, respectively. For the second (July to October 2021) and third (January to March 2022) study periods, we factored in the decreased usage of masks in public places as compared to the first study period. Specifically, we noted a decline of 54% in mask usage during the period from July to October 2021, and a decline of 48% from January to March 2022, relative to the period between November 2020 and February 2021, as documented by the COVID-19 Projections online database from the Institute of Health Metrics and Evaluation [25]. Consequently, we calculated the baseline values for the second and third study periods as 28.5% and 31.9%, respectively. The lower and upper bound estimates were estimated using the same approach.

The **probabilities of death** among individuals with COVID-19 infections (i.e., infection fatality ratio, IFR) in April 2020, July 2020, October 2020, January 2021, October 2021, February 2022, and November 2022 were obtained from published literature [26-30]. For the months where the IFR information was not available from the literature, we estimated IFRs from linear interpolation for months in between the available IFR estimates from the literature. The lower and upper bounds of the monthly probabilities of death were estimated using ranges of 75% to 125% of the baseline values.

To estimate the **probability of being infectious with COVID-19** (i.e., the fraction of population that was infectious at a given time), we used the following steps. First, we estimated the weekly incident COVID-19 infections using the period-specific IFRs and the number of deaths occurring three weeks later. This approach would assume an average time lag of approximately three weeks between COVID-19 infections and deaths. By integrating the IFR values reported in the literature [26-30] and the documented deaths associated with COVID-19 [31] throughout the study period, we derived an estimate of the number of COVID-19 infections. This approach was motivated by the recognition that COVID-19 infections may be underreported or under-detected due to various factors, including insufficient assessment of asymptomatic infections, testing resources, infrastructure limitations, financial constraints, or cultural factors [37, 38]. Consequently, mortality figures have been acknowledged as a more reliable indicator of the spread of COVID-19 cases [37]. Second, using the estimated weekly COVID-19 infections, we calculated the monthly prevalence of new COVID-19 infections among the total U.S. population [24]. Lastly, we calculated the fraction of the population that was infectious with COVID-19 at a given time, assuming an average infectious period of approximately five days [39]. Specifically, this involved dividing the monthly prevalence of new COVID-19 infections by 30-days (representing the number of days in a month) and subsequently multiplying the result by 5-days (representing the assumed duration of the infective period in days). The lower and upper bound estimates are subject to variations in IFR estimates.

The **probability of hospitalization** (hospitalization rates) associated with COVID-19 infection was estimated by dividing the reported number of new hospital admission [31] by the estimated number of COVID-19 infections one week prior, assuming a time difference of approximately one week between COVID-19 infections and hospitalizations. The lower and upper bound estimates depend on the variability of the IFR estimates. We also estimated the proportion of medically attended patients (i.e., non-hospitalized and hospitalized patients) by dividing the reported number of COVID-19 cases [31] by the estimated number of COVID-19 infections.**S2. Estimation of COVID-19 cases, hospitalizations, and deaths**

Using the estimated probability of COVID-19 infections applied to U.S. subway cars (estimated after adapting the Peng et al. model) and the estimated prevalence of infectious individuals in the United States, we estimated the monthly number of COVID-19 infections occurring during subway travel for each scenario, “Mask mandate” and “Mask recommendation.” This estimation involved four sequential steps: first, estimating the monthly number of subway cars carrying an infectious person in the United States, second, estimating the monthly number of passengers who rode on a subway car with an infectious passenger in the United States (potentially exposed), third, estimating the monthly number of non-immune passengers among those who have been potentially exposed, and fourth, estimating the monthly number of COVID-19 infections occurring on U.S. subways. The following formulas summarize the estimation.

1. Monthly number of subway cars carrying an infectious person = Total U.S. subway ridership per month × Probability of being infectious in the United States (i.e., prevalence of infectious individuals)
2. Monthly number of passengers potentially exposed to an infectious passenger in subway cars = Monthly number of subway cars carrying an infectious person × (Number of passengers per subway car ‒ 1)
3. Monthly number of non-immune and potentially exposed passengers = Monthly number of passengers potentially exposed to an infectious person × (1 ‒ Population immunity against COVID-19)
4. Monthly number of COVID-19 infections associated with exposures during subway rides = Monthly number of non-immune and potentially exposed passengers × Estimated probability of COVID-19 infection in a subway car from the adapted Peng et al. model

To estimate the monthly number of passengers potentially exposed to an infectious passenger in a subway car, we first estimated the monthly number of subway cars carrying an infectious person by multiplying the total U.S. subway ridership per month (obtained from the National Transit Database (NTD) [33]) by the monthly prevalence of infectious individuals with COVID-19 (shown in S1 Table) (step 1). We assumed that if a subway car had an infectious person, it would have exactly one infectious person. Therefore, certain subway cars had one infectious person per car, while the remaining subway cars would not have any infectious individuals and the passengers on these cars would not be potentially exposed. We believe this assumption is reasonable because the likelihood of encountering multiple infectious individuals in a subway car may be relatively low during the ongoing pandemic because the prevalence of infectious individuals in the United States remained relatively small relative to the number of passengers on each subway car.

Subsequently, we multiplied the estimated monthly number of subway cars carrying an infectious person by the number of passengers per subway car (excluding the infectious person) to estimate the monthly number of passengers exposed to an infectious passenger in a subway car (step 2). We then estimated the monthly number of non-immune and potentially exposed passengers by multiplying the estimated monthly number of passengers exposed to an infectious person by the ratio of population without COVID-19 immunity (i.e., 1 ‒ population immunity) (step 3). Finally, we estimated the monthly number of COVID-19 infections by multiplying the monthly number of non-immune and potentially exposed passengers by the estimated probability of COVID-19 infection in a subway car by month (step 4).

We also estimated the monthly number of hospitalizations resulting from infections that occurred on U.S. subways. We multiplied the monthly number of COVID-19 infections in a subway car (estimated from the step 4 above) by the estimated probability of COVID-19 hospitalization given infection (shown in S1 Table). To estimate the probability of COVID-19 hospitalization given infection (hospitalization rate), we divided the reported number of new hospital admission associated with COVID-19 [31] by the estimated number of COVID-19 infections one week prior, assuming a lag of approximately one week between infection and hospitalization. Using a similar approach, we estimated the monthly number of deaths arising from infections occurred on U.S. subways by multiplying the monthly number of COVID-19 infections in a subway car by the period-specific IFRs (shown in S1 Table).

**S3. Parameters used for estimating the costs of mask mandates**

**Table. Parameter values and sources**

| **Parameters** | **Unit** | **Baseline** | **LB** | **UB** | **Distribution** | **Reference** |
| --- | --- | --- | --- | --- | --- | --- |
| Reusable face mask (per count) ^a^ | $ | 4.1 | 0.6 | 16.7 | Gamma | [40] |
| Disposable face mask (per count) | $ | 0.3 | 0.1 | 2.0 | Gamma | [40, 41] |
| People wearing reusable face masks | % | 34 | 17 | 58 | beta-PERT | [42] |
| Disutility of wearing masks |  |  |  |  |  |  |
| Fraction experiencing disutility from wearing masks | % | 14 | 10 | 17 | beta-PERT | [43], assumption |
| WTP to ride on a subway car without a mask mandate for individuals with disutility | $ | 1.5 | 0.5 | 27.5 | Gamma | [43-45] |
| Communication/signage |  |  |  |  |  |  |
| Poster price (per count), (a) | $ | 58 | 35 | 138 | Gamma | [46] |
| Number of posters (per subway car), (b) | - | 3 | 1 | 6 | beta-PERT | Assumption |
| Number of subway cars (per city) |  |  |  |  |  |  |
| New York City, (c) | - | 6,455 | 6,455 | 6,455 | Constant | [47] |
| Chicago, (d) | - | 1,492 | 1,492 | 1,492 | Constant | [48] |
| Washington, D.C., (e) | - | 1,200 | 1,200 | 1,200 | Constant | [49] |
| All else cities, (f) | - | 1,614 | 1,453 | 1,776 | beta-PERT | Assumption |
| Total number of subway cars, (g)=(c)+(d)+(e)+(f) | - | 10,761 | 10,600 | 10,923 | N/A (calculated) |  |
| Total number of posters (h)=(b)×(g) | - | 32,283 | - | - | N/A (calculated) |  |
| Total costs of posters in the first month ($), (i)=(a)×(h) | $ | 1,856,273 | - | - | N/A (calculated) |  |
| Total costs of poster in subsequent months ($), (j)=(i)×25% | $ | 464,068 | - | - | N/A (calculated) |  |
| COVID-19 treatment (per person) |  |  |  |  |  |  |
| Non-hospitalized patient | $ | 1,008 | 927 | 3,045 | Gamma | [50-52] |
| Hospitalized patient | $ | 24,826 | 19,934 | 41,611 | Gamma | [53-55] |
| Death | $ | 27,017 | 25,527 | 32,015 | Gamma | [53, 56] |

^a^ Reusable masks were assumed to be used for one month.

WTP, willingness-to-pay; LB, lower bound; UB, upper bound; PERT, program evaluation and review technique

The **costs of purchasing face masks** (reusable and disposable masks) were based on a previous study [41] and Amazon marketplace [40]. The baseline estimates were the average price per count for reusable and disposable face masks, respectively, and the lower and upper bound estimates represented the minimum and maximum prices. To estimate the monthly costs of purchasing reusable face masks among U.S. subway passengers, we initially estimated the number of unique passengers per month using the NTD monthly subway ridership data, assuming that individuals would use reusable masks for a month. We divided the NTD monthly ridership data by 80 (2×2×20), assuming that, on average, each passenger had one transfer per trip, would complete one round trip each day, and used subways for 20 days per month (corresponding to working days). We made this assumption due to the nature of the NTD ridership data, which counted passengers each time they boarded a vehicle, regardless of the number of vehicles used for their journey from origin to destination (i.e., unlinked passenger trips) [33]. Subsequently, we multiplied the estimated number of unique passengers per month by the average price per count for reusable masks and the proportion of people wearing reusable masks. The estimated **proportion of people wearing reusable masks** was based on a previous survey study [42]. According to the study, on average, 34% of face masks worn by the public were cloth masks, with a range of 17% to 58% [42]. We used 34% as the baseline estimate, with the lower and upper bound estimates representing the range observed in the study.

For the monthly costs of purchasing disposable masks, we estimated the monthly number of disposable masks used among U.S. subway passengers by dividing the NTD monthly ridership data by three (2×1.5). We assumed that each passenger had one transfer per trip and would complete one round tip each day, while some passengers would use one disposable mask for a day (i.e., they would continue to use the same disposable masks for round trips). We then multiplied the estimated monthly usage of disposable masks by the average price per unit for disposable masks and the proportion of people wearing disposable face masks (i.e., 1 ‒ Proportion of people wearing reusable face masks [42]).

Finally, we estimated the incremental monthly costs of purchasing masks under “Mask mandate” by multiplying the estimated monthly costs of purchasing reusable and disposable masks by the proportions of individuals who would wear masks due to the mask mandate (i.e., people who would not wear masks in the absence of such a mandate).

We estimated **passengers’ disutility from compliance** for “Mask mandate” by estimating the opportunity costs for individuals who prefer not to wear masks. These opportunity costs were estimated based on a previous survey study that examined respondents’ willingness-to-pay (WTP) extra to switch to an airline with a different mask policy [43]. The study found that 14% of participants were willing to pay more to switch from a mask mandate flight to a non-mask mandate flight, with an average WTP amount of 27.8% of roundtrip airfare [43]. These average estimates were used as the baseline for our calculations. There might be potential utility for individuals who would feel more comfortable if other passengers were wearing masks and would derive utility from the mask mandate. Thus, the disutility to the individuals opposed to mask wearing may be offset. However, we did not account for these benefits in this analysis to be conservative.

We assumed that 14% of subway passengers would prefer not to wear masks under “Mask mandate” and would derive disutility from the mandate for our baseline analysis. The lower and upper bound estimates were assumed to be a range of 75% to 125% of the baseline values. Our estimate of the concomitant WTP to avoid wearing a mask on a subway trip was $1.5 (27.8% × $2.75 × 2), which was based on a one-way subway fare of $2.75 in New York City [44]. We estimated a lower bound of $0.5 (10% × $2.25 × 2), taking into account that the lowest estimate in the study was 10% [43], and we used a fare of $2.25 based on the subway fare in Washington D.C. [45]. The upper bound was assumed to be ten times the one-way subway fare ($2.75 × 10). This large range was used to account for the uncertainty in transferring the WTP amount from aircraft to subway passengers.

We assumed that there were **posters or signs** (e.g., interior car cards) displayed in subway cars under the “Mask mandate” scenario, emphasizing the requirement for passengers to wear masks while on board. We assumed an average of three posters per subway car, with a range of one to six posters. The cost of each poster was estimated based on printing expenses obtained from FedEx Office, which amounted to $29 per poster sized 22"×28" [46], with price ranges varying from $17 to $69 depending on the poster size [46]. We also multiplied the poster price by two, assuming that there might be potential additional costs for administrative tasks such as installation, shipping, or labor, etc. To estimate the total number of posters required, we calculated the total number of subway cars in the United States. The number of subway cars in major cities such as New York City, Chicago, and Washington D.C. were used to estimate the total number of cars. Since these three cities accounted for an average of approximately 85% of the total ridership [33], we assumed that they also represented 85% of the total subway cars in the United States. Consequently, we assumed the remaining cities accounted for 15% of the total subway cars and used this ratio to estimate the baseline number of subway cars in those remaining cities. The lower and upper bound estimates were applied only to the remaining cities and were assumed to have a range of ±10% of the baseline values. Finally, we estimated the total cost of the posters by multiplying the price per poster by the total number of posters required (total number of subway cars in the United States × number of posters per subway car). The total costs of posters or signs in subway cars were considered as startup costs and applied in the first month of the analysis (November 2020). We also assumed that 25% of the startup costs would be incurred in subsequent months as maintenance costs. We did not attempt to account for any enforcement costs for the “Mask mandate” scenario.

The **costs associated with COVID-19 treatment** were estimated for each scenario, “Mask mandate” and “Mask recommendation”. The treatment costs were based on the average estimated per-patient costs reported in published literature and were estimated by disease severity: non-hospitalized (outpatient visits only), hospitalized, and death. To maintain a conservative approach, non-medically attended patients were not considered, given the relatively lower costs of obtaining over-the-counter drugs to alleviate COVID-19 related symptoms. The proportion of medically attended patients (non-hospitalized and hospitalized) was estimated by dividing the reported number of COVID-19 cases [31] by the estimated number of COVID-19 infections that we calculated using IFRs and reported deaths. The baseline estimate for the treatment cost of outpatients ($1,008 per person) was based on the FAIR Health COVID-19 Cost Tracker [50]. The lower and upper bound estimates ($927 - $3,045 per person) were from Weiner et al. utilizing the Blue health intelligence data [51], and from Bartsch et al. through a simulation modeling study [52], respectively. For hospitalized patients, the baseline costs ($24,826 per person) were obtained from Shrestha et al. who used the Premier Healthcare Database Special COVID-19 Release (PHD-SR) [53]. The lower bound estimate ($19,934 per person) was sourced from Ohsfeldt et al. who used the PHD-SR data [54], while the upper bound estimate ($41,611 per person) was derived from the Peterson-KFF Health System Tracker using the MarketScan data [55]. For patients who died while infected, the baseline ($27,017 per person) and lower bound estimates ($25,527 per person) were from Shrestha et al. [53]. The upper bound estimate ($32,015 per person) was from Tsai et al. utilizing Medicare fee-for-service administrative claims data [56].

**S4. Sensitivity analyses on the fraction of population wearing masks with mask mandates**

We are uncertain that individuals opposed to a mask mandate would continue to wear masks the longer a pandemic persists. If mask mandates are not strictly enforced, which is likely to occur on subways, the fraction of the population wearing masks with the mandate in effect may decline over time as we previously assumed for the fraction of the population who would wear masks in the “Mask recommendation” scenario. We conducted additional sensitivity analyses around this possibility. Specifically, our base assumption for “Mask mandate” is that most people would wear masks (baseline 95%, ranging from 90% to 100%). Here, we explored a second scenario in which only half of the people who would not choose to wear masks if recommended would actually wear masks with a mandate. The proportions were estimated at 81% in the Alpha, Beta, and Gamma period (compared to 62% who wear masks if recommended), 64% in the Delta period (compared to 29%), and 66% in the early Omicron period (compared to 32%). For example, for the Alpha, Beta, and Gamma period, 81% was calculated using the following equation: 61.8% + 0.5 × 38.2% = 81%. Additionally, for this analysis, we also assumed that disutility costs would be lower (50% of the base case analysis) since the fraction of the population most opposed to wearing masks would be less likely to wear masks in this comparison. This approach provides additional context around the potential incremental cost-effectiveness ratio (ICER) of our ”Mask mandate” scenario if mask wearing is lower than expected and proportional to rates of mask wearing under the “Mask recommendation” scenario.

When we used lower proportions of people wearing masks for the “Mask mandate” scenario, which were proportional to the rates assumed for passengers who wear masks under “Mask recommendation” during the three periods (81%, 64%, and 66%, respectively), the ICER estimates were still lower than the HHS-recommended WTP of $11.4 million per averted death, with cost savings observed in January and February in 2022 (S1 Table). Additionally, we found that the ICER estimates remained below $11.4 million per averted death, even when considering a slight increase in coverage rates for mask-wearing under “Mask mandate”, in comparison to the rates for “Mask recommendation” across the three different periods. These rates were 62.9% with an ICER of $10.5 million in November 2020, 28.8% with an ICER of $11.1 million in July 2021, and 31.92% with an ICER of $9.1 million in January 2022 (S4 Fig).

**S5. Sensitivity analysis on the estimation of COVID-19 prevalence in subway passengers**

Our analysis uses national average estimates for the COVID-19 infection fatality ratio (IFR), deaths, and hospitalization data. If the IFR or hospitalization rate are much different in rural versus urban areas, this may bias our results. In this section, we used COVID-19 IFR, deaths, and hospitalization data specific to the U.S. metropolitan areas, in contrast to the national estimates used in the main analyses. This information was used to estimate the number of infectious subway passengers, along with hospitalized patients and deaths given infections.

We identified metropolitan areas at the county level based on the urban-rural classification scheme for counties by the National Center for Health Statistics (NCHS) [57]. This scheme comprises six levels: four metropolitan (large central metro, large fringe metro, medium metro, and small metro) and two nonmetropolitan (micropolitan and noncore). We specifically included counties corresponding to the four metropolitan levels.

The COVID-19 IFR estimates specific to metropolitan areas were calculated by multiplying the national estimates of IFR used in the main analyses by the weighted odds ratios of mortality rates for rural dwellers with COVID-19. These adjusted IFR estimates by month for metropolitan areas were calculated using results from a previous study that reported an adjusted odds ratio of 1.36 for mortality rates among rural residents with COVID-19 compared to urban residents [58], considering county population [59], and using the NCHS urban-rural scheme for counties [57]. COVID-19 deaths in metropolitan counties were determined based on weekly deaths by county [60] and the NCHS urban-rural scheme for counties [57]. Hospitalizations specific to metropolitan areas were based on facility-level weekly inpatient data associated with COVID-19 [61], with metropolitan facilities identified using the NCHS urban-rural county-level scheme [57].

When we used metropolitan-specific estimates of IFR, hospitalizations, and deaths, the ICER estimates for mask mandates were comparable to the main analysis results (S3 table). These ranged from cost-savings in the early Omicron period to $3.0 million per death averted during the Alpha, Beta, and Gamma period, consistent with the main analysis results.

**S1 Fig. Full results of one-way sensitivity anlaysis for the cost-effectiveness of ”Mask mandate” relative to “Mask recommendation” on subways**

1. Alpha, Beta, Gamma period (November 2020‒Feburary 2021)


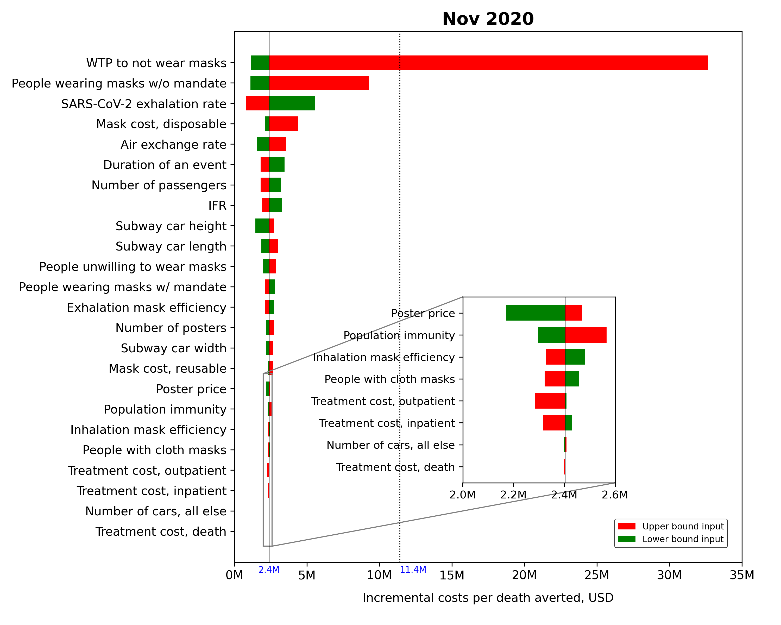

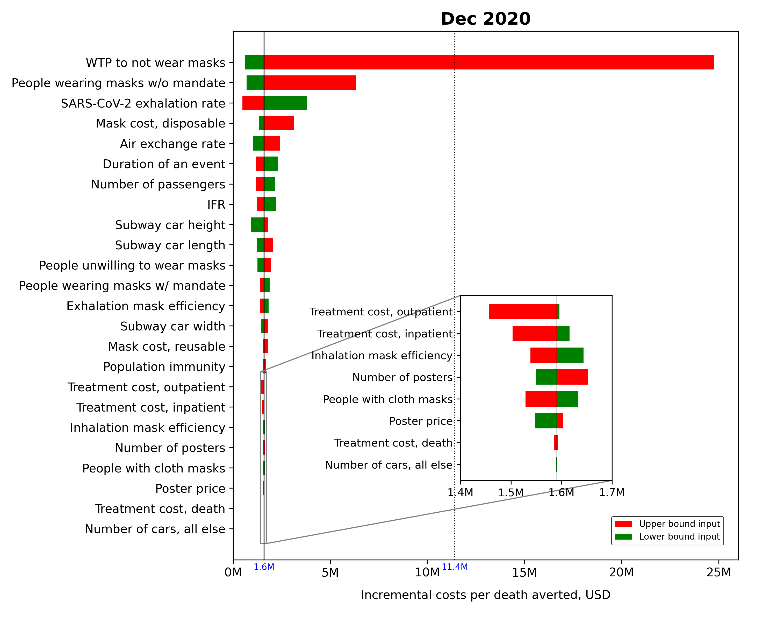


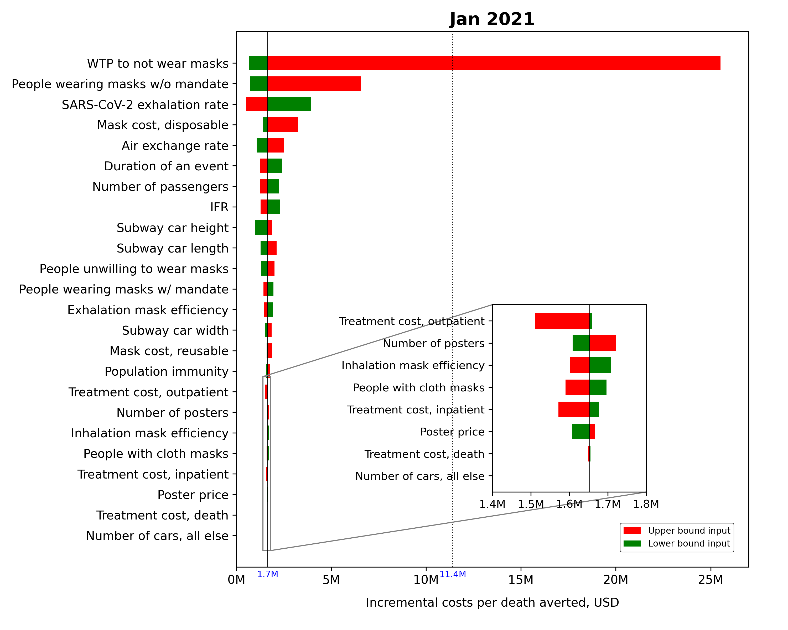

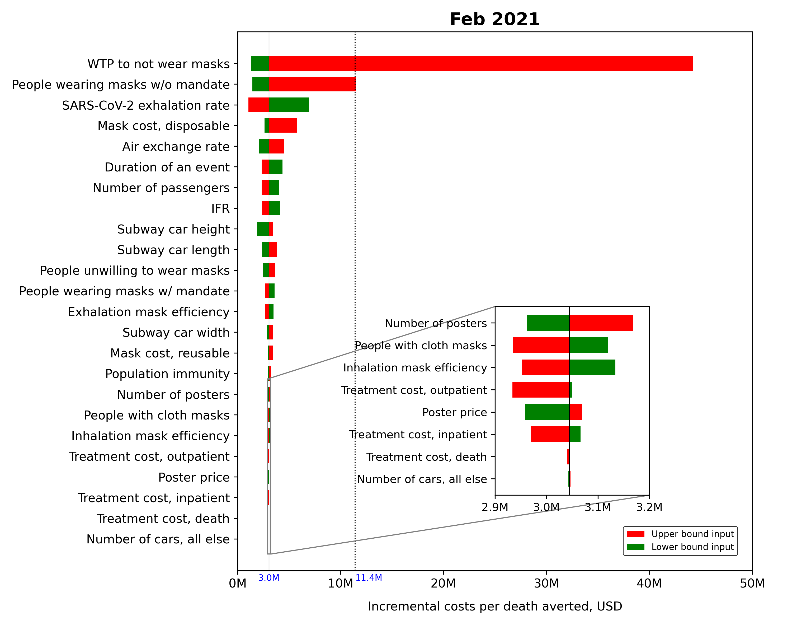


1. Delta period (July‒October 2021)


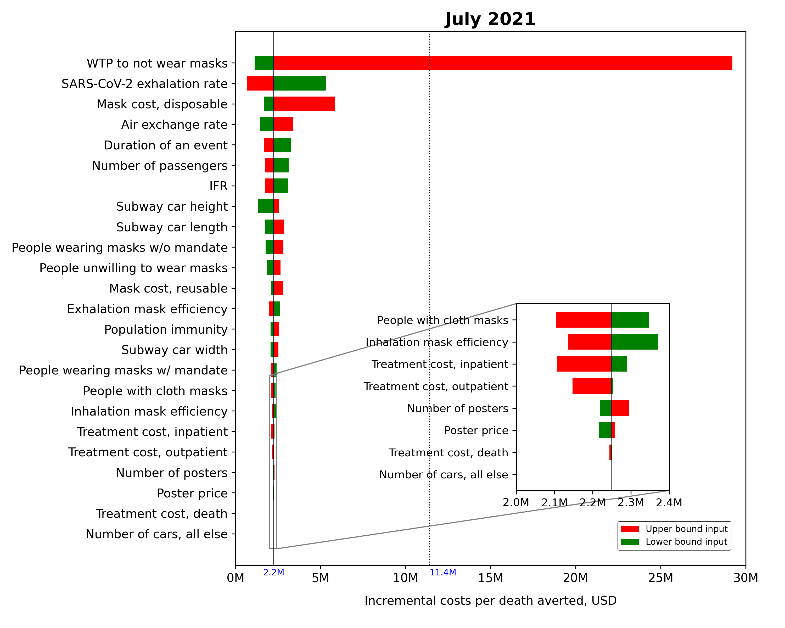

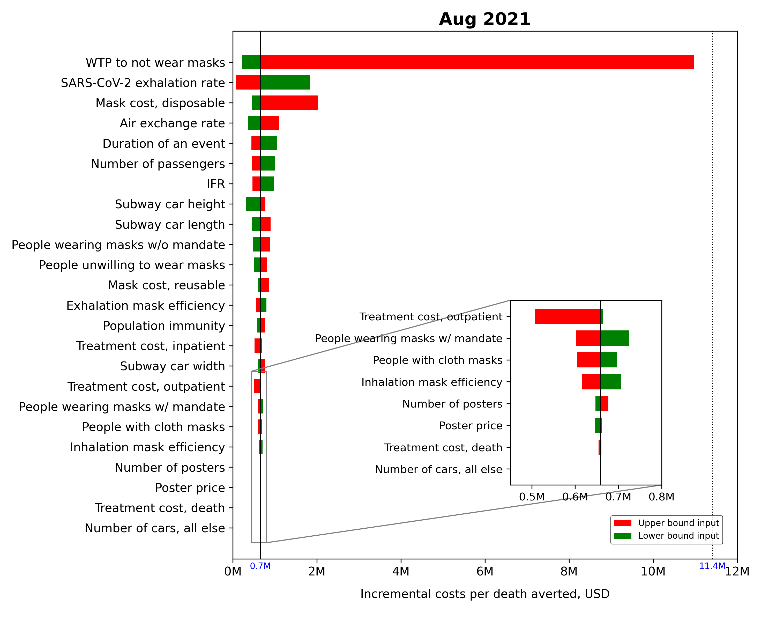


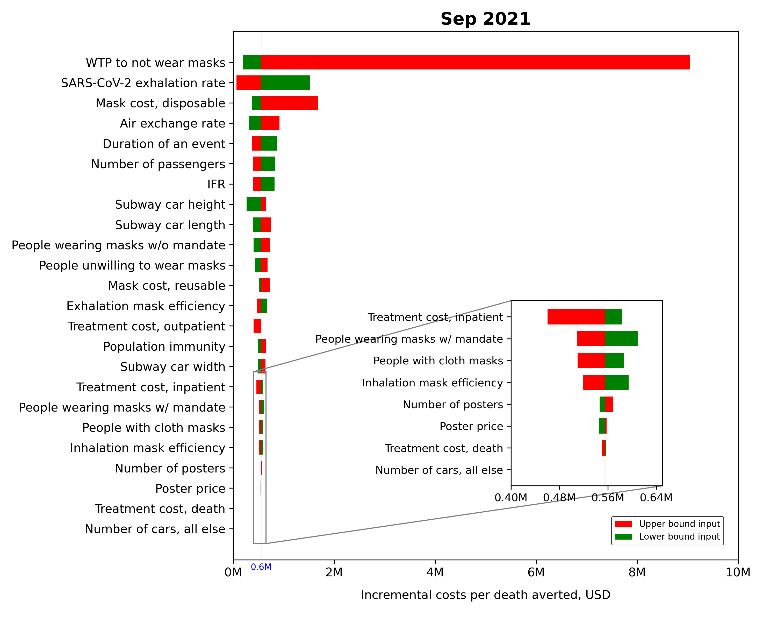

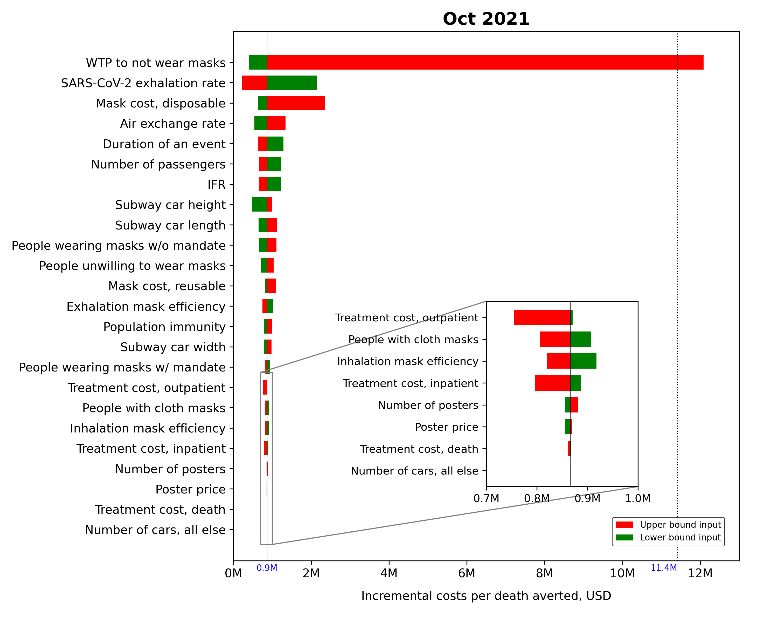


1. Early Omicron period (January – March 2022)


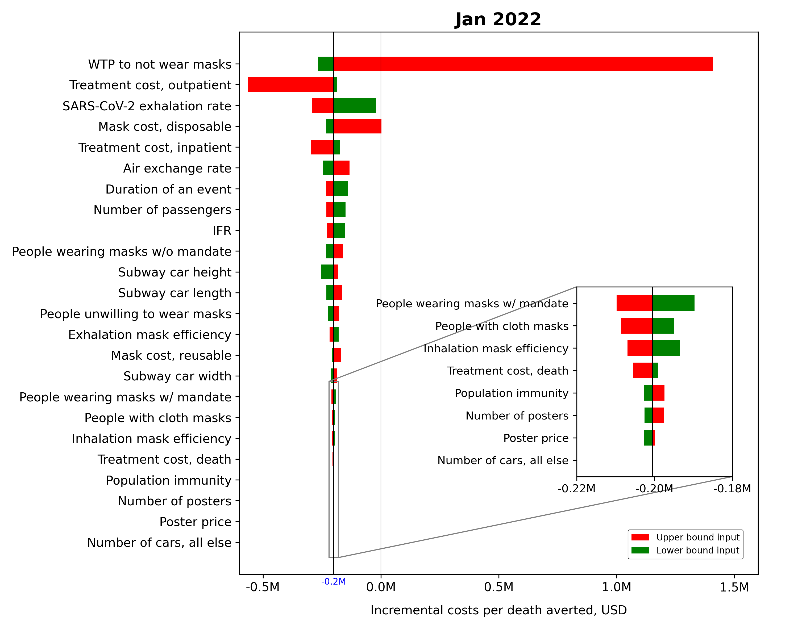

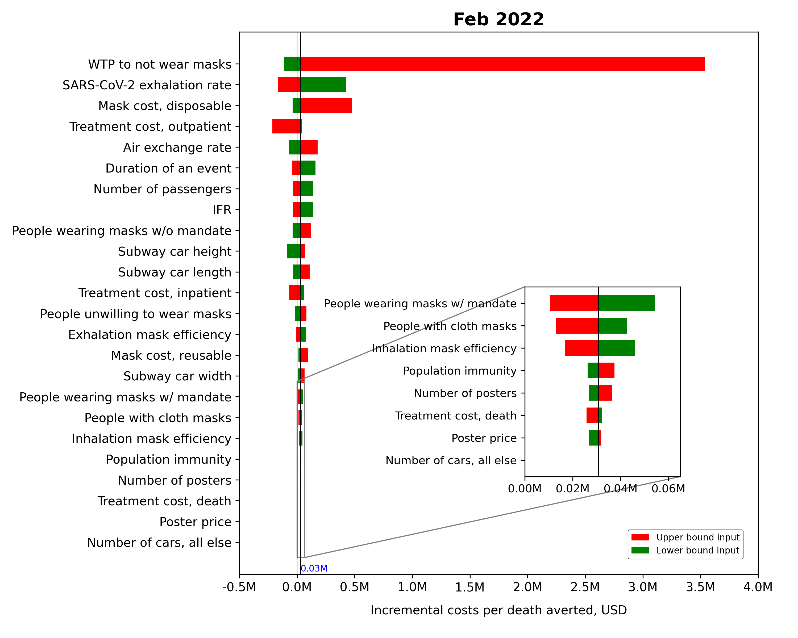


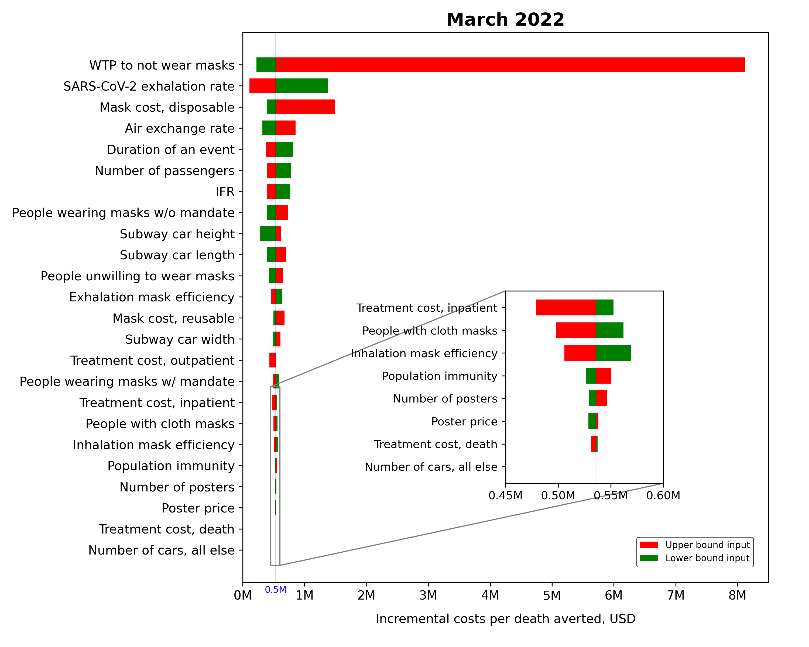


(a) Alpha, Beta, Gamma period, (b) Delta period, and (c) Early Omicron period. The solid lines represent the baseline incremental costs per death averted (i.e., ICER) and the dotted lines indicate the cost-effectiveness threshold (i.e., central value of the value per statistical life, $11.4 million). Each horizontal bar illustrates the changes in the ICER as each input parameter is varied over the uncertainty range of the lower and upper bound estimates while maintaining all other parameters at their base case values.

**S2 Fig. Probabilistic sensitivity analysis results, incremental cost per death averted scatter plots, for the cost-effectiveness of “Mask mandate” relative to “Mask recommendation” on subways**

1. Alpha, Beta, Gamma period (November 2020‒Feburary 2021)


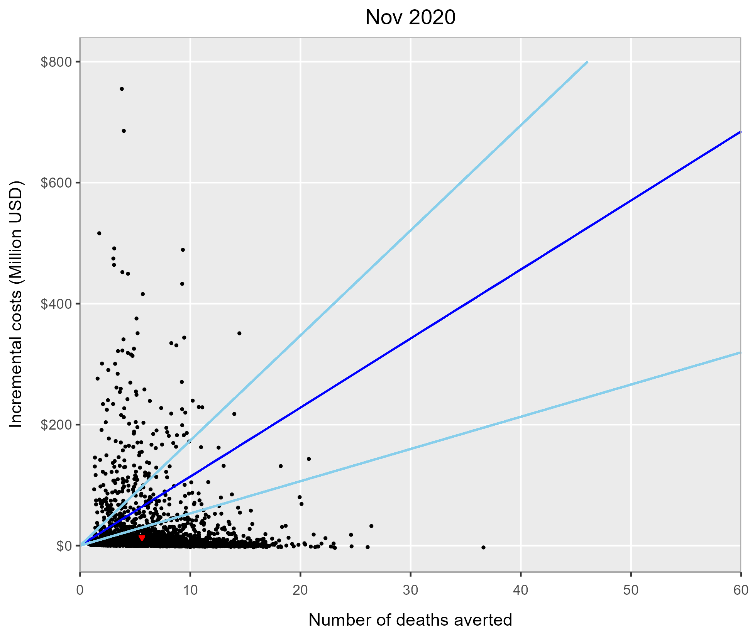

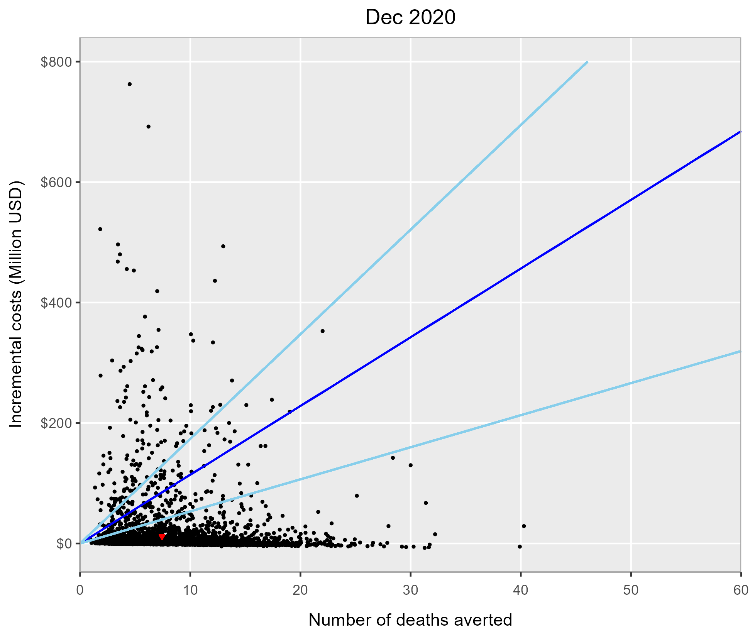


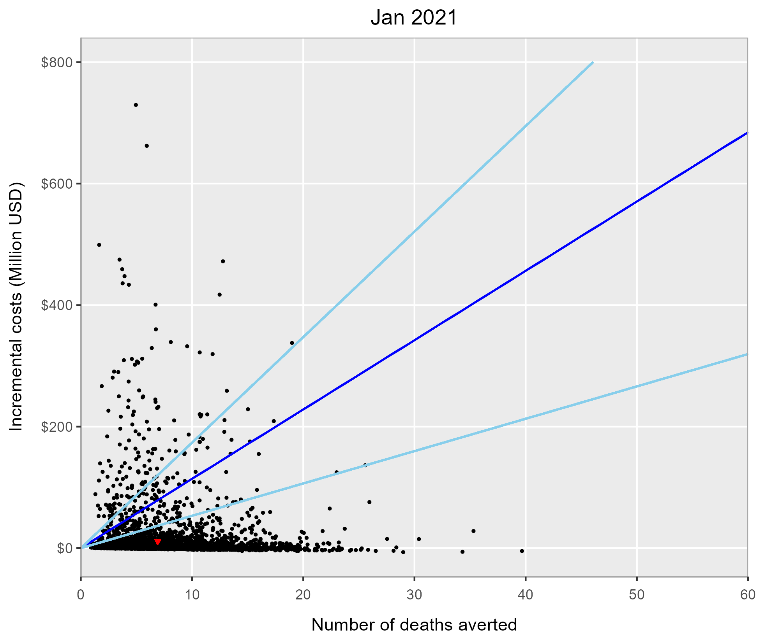

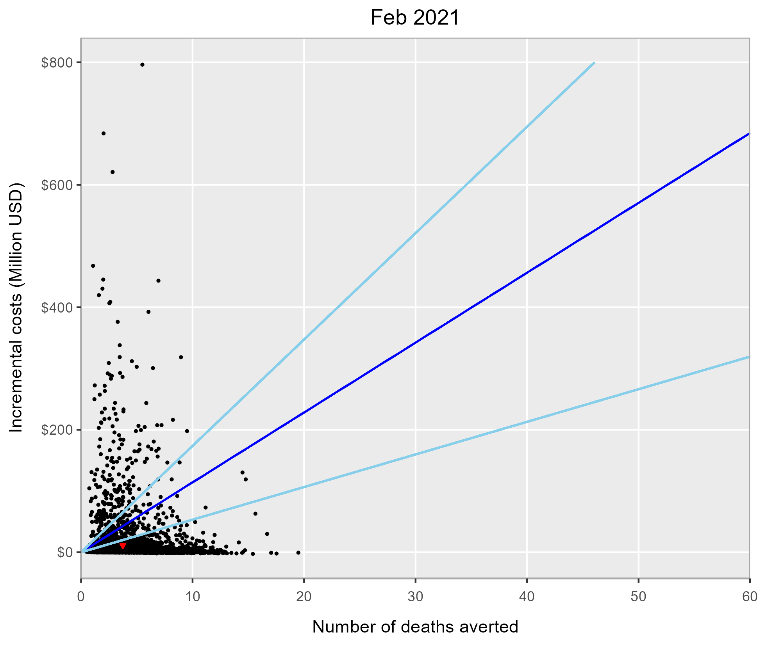


1. Delta period (July‒October 2021)


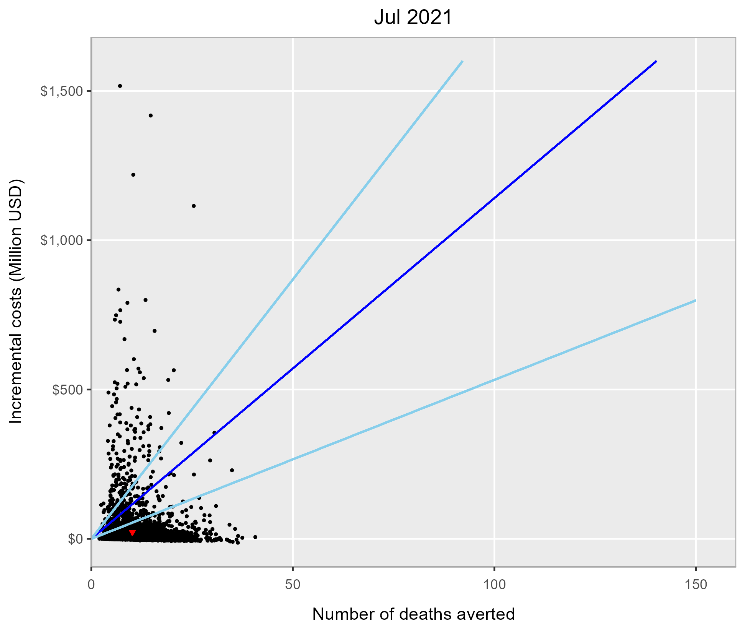

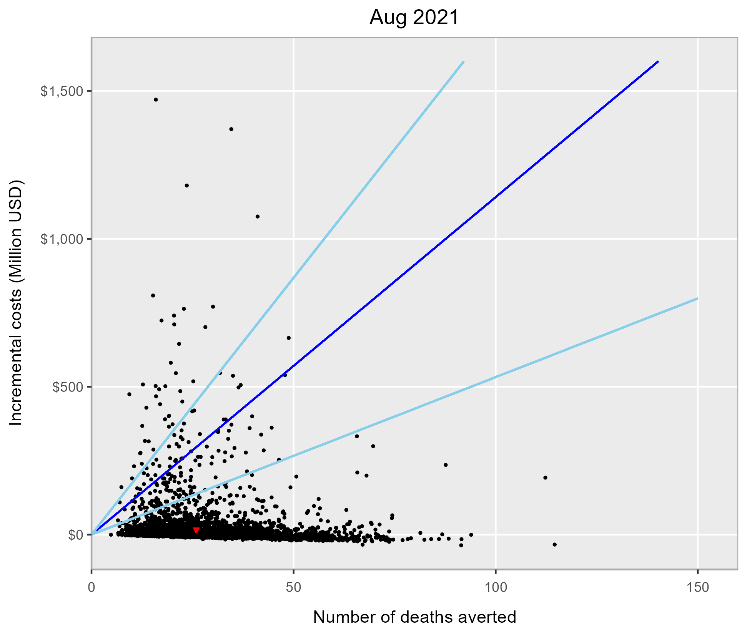


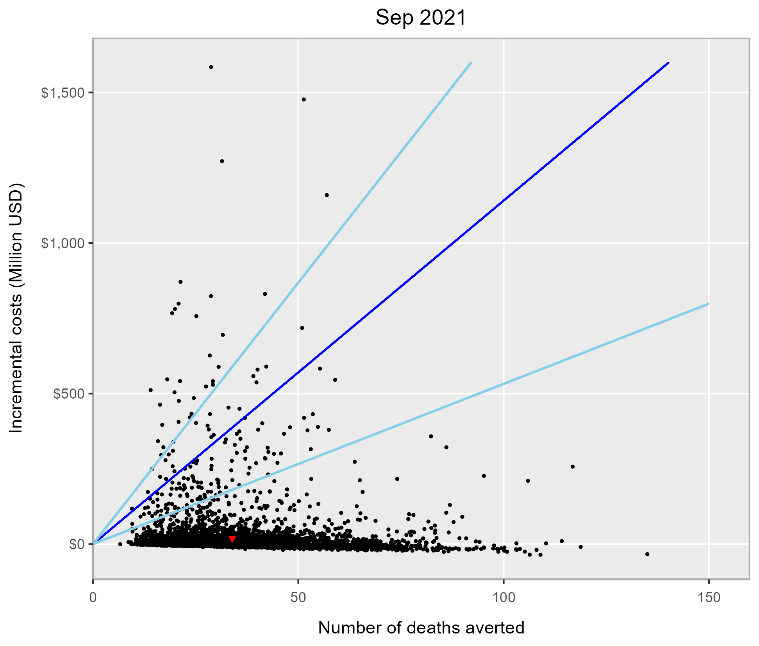

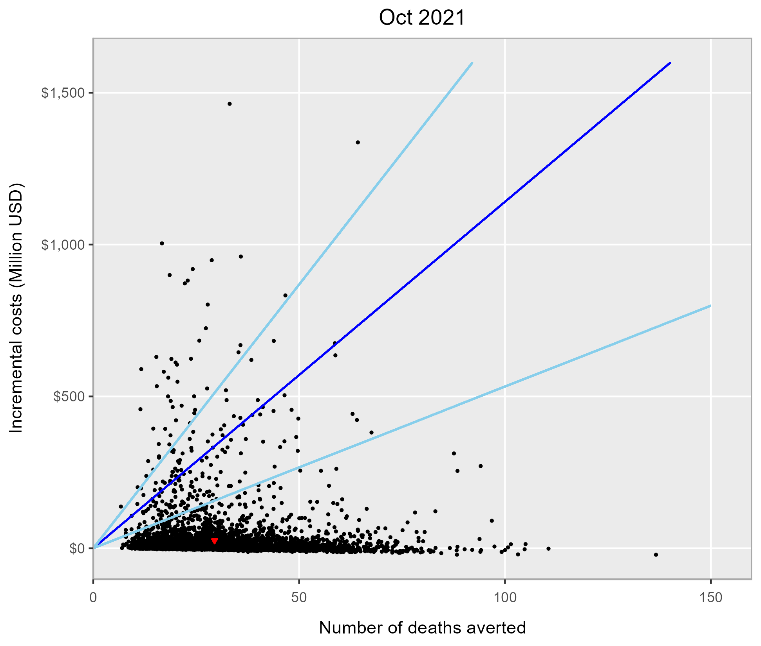


1. Early Omicron period (January‒March 2022)


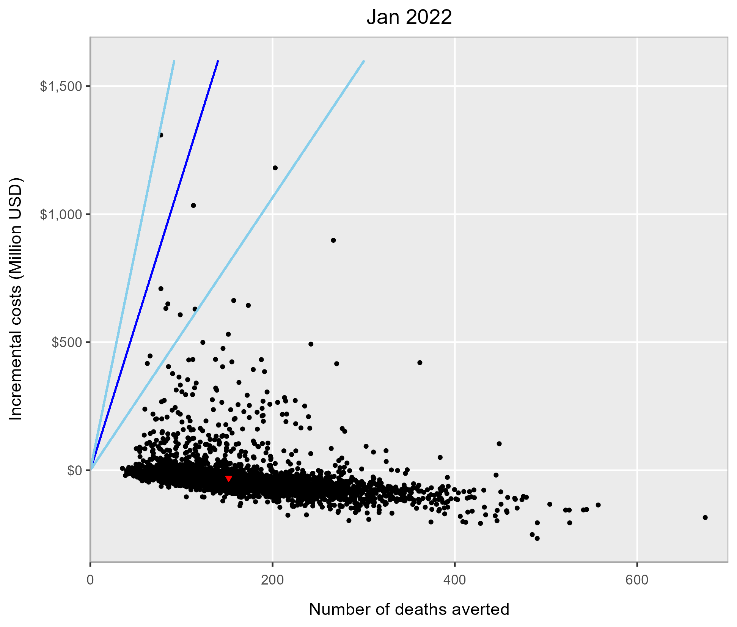

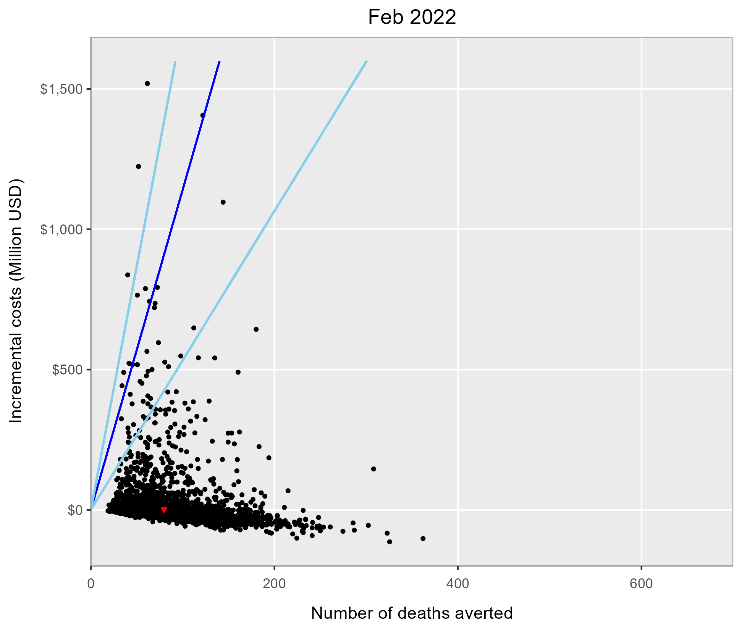


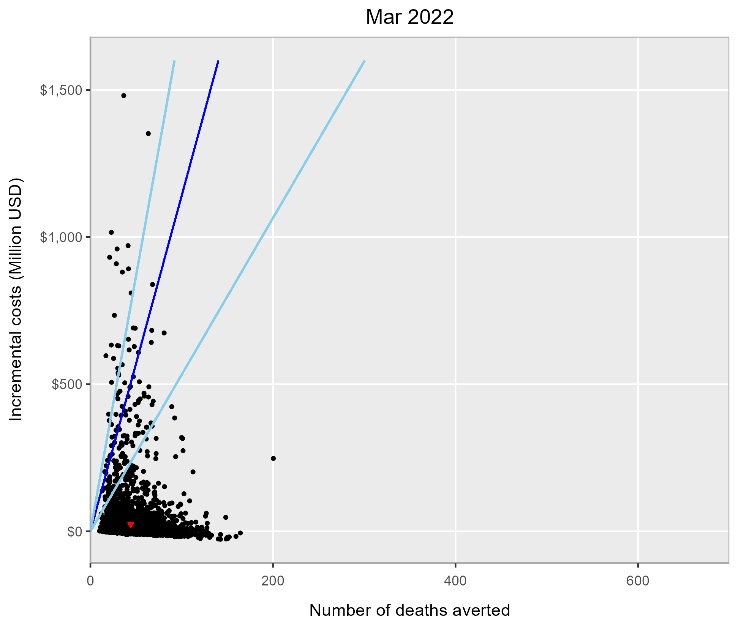


(a) Alpha, Beta, Gamma period, (b) Delta period, and (c) Early Omicron period. The blue lines indicate the central value of the value per statistical life (VSL), which was set at $11.4 million in 2020 USD, serving as the threshold for determining the cost-effectiveness of the intervention. The sky blue lines represent the lower bound VSL estimate (right; $5.3 million) and the upper bound VSL estimate (left; $17.4 million), respectively. Red downward-pointing triangles indicate the base-case analyses results for each month.

The interpretation of the plot reveals that the proportion of dots to the right of the diagonal blue line, representing the willingness-to-pay of $11.4 million per death averted, is as follows: 95% (Nov 2020), 96% (Dec 2020), 96% (Jan 2021), 94% (Feb 2021), 95% (July 2021), 98% (Aug 2021), 99% (Sep 2021), 98% (Oct 2021), 99.9% (Jan 2022), 99.8% (Feb 2022), and 99.0% (Mar 2022). These proportions imply the likelihood that the “Mask mandate” scenario is cost-effective under the threshold of $11.4 million per averted death.

**S3 Fig. Incremental cost per death averted (i.e., ICER) estimates by the number of infectious persons per subway car**

We estimated the ICERs (measured as the incremental costs per death averted) by varying the number of infectious persons per subway car based on the following assumptions: (1) the maximum number of infectious persons per subway car would be 20% of the total number of passengers per subway car (i.e., 4 infectious persons from November 2020 to February 2021), 7 infectious persons from July 2021 to October 2021, and 12 infectious persons in Jan 2022, February 2022, and March 2022, respectively), and (2) the number of infectious persons was assumed to be the same for each subway car. To maintain the same prevalence assumptions among passengers on subway cars, we re-calculated the number of subway cars with an infectious individual for each scenario. All estimates present the ICER estimates for the “Mask mandate” scenario relative to the “Mask recommendation” scernario. ICER, incremental cost-effectiveness ratio

**S1 Table. Estimated incremental health outcomes and costs of mask mandates on subways with lower proportions of people wearing masks with a mandate (81% in the Alpha, Beta, and Gamma period, 64% in the Delta period, and 61% in the early Omicron period) for the “Mask mandate” scenario relative to the “Mask recommendation” scenario**

|  | **Incremental health outcomes relative to “Mask recommendation”** | | | **Incremental costs relative to “Mask recommendation” (Thousand USD)** | | | **ICER**  **(Thousand USD/death averted)** |
| --- | --- | --- | --- | --- | --- | --- | --- |
|  | COVID-19 infections averted | Hospitalizations averted | Deaths averted | Intervention costs | Treatment costs | Total  costs |  |
| **Alpha, Beta, Gamma period** |  |  |  |  |  |  |  |
| Nov 2020 | 431 | 18 | 3 | $8,502 | -$724 | $7,778 | $2,315 |
| Dec 2020 | 580 | 23 | 4 | $7,196 | -$990 | $6,206 | $1,398 |
| Jan 2021 | 547 | 20 | 4 | $6,903 | -$897 | $6,006 | $1,454 |
| Feb 2021 | 299 | 10 | 2 | $6,495 | -$430 | $6,065 | $2,720 |
| **Delta period** |  |  |  |  |  |  |  |
| Jul 2021 | 821 | 50 | 6 | $13,506 | -$1,688 | $11,819 | $2,007 |
| Aug 2021 | 2,105 | 126 | 15 | $13,143 | -$4,656 | $8,487 | $567 |
| Sep 2021 | 2,776 | 111 | 20 | $14,130 | -$4,759 | $9,371 | $478 |
| Oct 2021 | 2,433 | 71 | 17 | $16,142 | -$3,155 | $12,988 | $763 |
| **Early Omicron period** |  |  |  |  |  |  |  |
| Jan 2022 | 30,530 | 504 | 88 | $11,841 | -$30,667 | -$18,826 | Cost saving |
| Feb 2022 | 30,748 | 270 | 46 | $13,488 | -$13,501 | -$13 | Cost saving |
| Mar 2022 | 18,080 | 87 | 26 | $16,057 | -$4,137 | $11,920 | $467 |

ICER, incremental cost-effectiveness ratio

**S4 Fig. Net cost per averted death (i.e., incremental cost-effectiveness ratio, ICER) versus the fraction of people wearing masks for the “Mask mandate” scenario relative to the “Mask recommendation” scenario**

1. November 2020 (Alpha, Beta, and Gamma period)
2. July 2021 (Delta period)
3. January 2022 (early Omicron period)

Dotted blue line (vertical) represents the proportion of people wearing masks under the “Mask recommendation” scenario. Dotted orange line (horizontal) represents the HHS-recommended willingness-to-pay of $11.4 million per averted death, which was used as a threshold to determine whether the intervention is cost-effective.

**S2 Table. Estimated incremental health outcomes and costs of mask mandates on subways under the assumption that no people would wear masks for the “Mask recommendation” scenario**

|  | **Incremental health outcomes relative to “Mask recommendation”** | | | **Incremental costs relative to “Mask recommendation” (Thousand USD)** | | | **ICER**  **(Thousand USD/death averted)** |
| --- | --- | --- | --- | --- | --- | --- | --- |
|  | COVID-19 infections averted | Hospitalizations averted | Deaths averted | Intervention costs | Treatment costs | Total  costs |  |
| **Alpha, Beta, Gamma period** |  |  |  |  |  |  |  |
| Nov 2020 | 2,405 | 98 | 19 | $20,006 | -$4,039 | $15,967 | $853 |
| Dec 2020 | 3,236 | 129 | 25 | $18,847 | -$5,522 | $13,325 | $537 |
| Jan 2021 | 3,051 | 112 | 23 | $18,049 | -$5,007 | $13,042 | $567 |
| Feb 2021 | 1,671 | 56 | 13 | $16,935 | -$2,403 | $14,532 | $1,163 |
| **Delta period** |  |  |  |  |  |  |  |
| Jul 2021 | 2,171 | 131 | 16 | $29,796 | -$4,462 | $25,334 | $1,625 |
| Aug 2021 | 5,565 | 333 | 40 | $28,980 | -$12,310 | $16,669 | $421 |
| Sep 2021 | 7,340 | 293 | 52 | $31,199 | -$12,583 | $18,617 | $359 |
| Oct 2021 | 6,431 | 187 | 45 | $35,725 | -$8,341 | $27,385 | $608 |
| **Early Omicron period** |  |  |  |  |  |  |  |
| Jan 2022 | 85,733 | 1,414 | 246 | $26,522 | -$86,117 | -$59,595 | Cost saving |
| Feb 2022 | 86,343 | 758 | 130 | $30,293 | -$37,913 | -$7,620 | Cost saving |
| Mar 2022 | 50,771 | 244 | 72 | $36,177 | -$11,617 | $24,560 | $343 |

ICER, incremental cost-effectiveness ratio

**S3 Table. Estimated incremental health outcomes and costs of mask mandates on subways using hospitalization, death, and population data that are more specific to metropolitan areas for the “Mask mandate” scenario relative to the “Mask recommendation” scenario**

|  | **Incremental health outcomes relative to “Mask recommendation”** | | | **Incremental costs relative to “Mask recommendation” (Thousand USD)** | | | **ICER**  **(Thousand USD/death averted)** |
| --- | --- | --- | --- | --- | --- | --- | --- |
|  | COVID-19 infections averted | Hospitalizations averted | Deaths averted | Intervention costs | Treatment costs | Total  costs |  |
| **Alpha, Beta, Gamma period** |  |  |  |  |  |  |  |
| Nov 2020 | 660 | 34 | 5 | $14,720 | -$1,371 | $13,349 | $2,730 |
| Dec 2020 | 953 | 48 | 7 | $13,494 | -$1,957 | $11,537 | $1,658 |
| Jan 2021 | 931 | 43 | 7 | $12,928 | -$1,820 | $11,108 | $1,660 |
| Feb 2021 | 522 | 23 | 4 | $12,138 | -$901 | $11,238 | $3,021 |
| **Delta period** |  |  |  |  |  |  |  |
| Jul 2021 | 1,125 | 103 | 8 | $25,857 | -$3,358 | $22,499 | $2,926 |
| Aug 2021 | 3,964 | 253 | 27 | $25,150 | -$9,317 | $15,833 | $589 |
| Sep 2021 | 4,567 | 236 | 31 | $27,072 | -$9,699 | $17,372 | $566 |
| Oct 2021 | 3,860 | 162 | 26 | $30,990 | -$6,594 | $24,396 | $948 |
| **Early Omicron period** |  |  |  |  |  |  |  |
| Jan 2022 | 52,195 | 951 | 143 | $22,605 | -$59,639 | -$37,034 | Cost saving |
| Feb 2022 | 49,917 | 547 | 71 | $25,810 | -$26,713 | -$903 | Cost saving |
| Mar 2022 | 29,056 | 216 | 39 | $30,809 | -$9,039 | $21,770 | $558 |

ICER, incremental cost-effectiveness ratio

**References**

1. Peng Z, Rojas ALP, Kropff E, Bahnfleth W, Buonanno G, Dancer SJ, et al. Practical Indicators for Risk of Airborne Transmission in Shared Indoor Environments and Their Application to COVID-19 Outbreaks. Environmental Science & Technology. 2022;56(2):1125-37. doi: 10.1021/acs.est.1c06531.

2. Metrorail Fleet Management Plan: Washington Metropolitan Area Transit Authority; 2021 [April 21, 2023]. Available from: <https://www.wmata.com/initiatives/plans/upload/Metrorail-Fleet-Management-Plan.pdf>.

3. The MBTA Vehicle Inventory Page [cited 2023 February 16]. Available from: <http://roster.transithistory.org/>.

4. Boston LRV: Ansaldobreda SpA; [cited 2023 February 16]. Available from: <https://trampicturebook.de/tram/download/ansaldobreda/BostonLightRail.pdf>.

5. New York City Subway Current Fleet [cited 2022 October 31]. Available from: <https://www.nycsubway.org/wiki/Current_Fleet>.

6. CTA Car Roster [cited 2023 February 16]. Available from: <https://www.chicago-l.org/trains/roster/>.

7. Burd C, Burrows M, McKenzie B. Travel Time to Work in the United States: 2019. American Community Survey Reports [updated March 2021April 3, 2023]. Available from: <https://www.census.gov/content/dam/Census/library/publications/2021/acs/acs-47.pdf>.

8. Schwartz S. Public Transit and COVID-19 Pandemic: Global Research and Best Practices. Sam Schwartz Consulting, 2020 September. Report No.

9. Transport Chicago 2020 - Keynote Address: Sam Schwartz. 2020.

10. How's The Air In There? A Look At Ventilation On The MBTA: wbur; 2020 [updated September 28; cited 2022 November 8]. Available from: <https://www.wbur.org/news/2020/09/28/mbta-ventilation>.

11. Mayor Lightfoot Announces CTA To Provide Rear Door Boarding, New System to Reduce Crowding on All Buses: City of Chicago; 2020 [updated April 9; cited 2023 April 11]. Available from: <https://www.chicago.gov/city/en/depts/mayor/press_room/press_releases/2020/april/BusRearDoorEntry.html>.

12. Sutter L. CTA Increases Capacity Limits on Buses and Trains: NBC Chicago; 2021 [updated March 28; cited 2023 April 11]. Available from: <https://www.nbcchicago.com/news/local/cta-increases-capacity-limits-on-buses-and-trains/2473143/>.

13. Freishtat S. CTA ends capacity limits on trains, buses as Chicago reopens: Chicago Tribune; 2021 [updated Jun 11; cited 2023 April 11]. Available from: <https://www.chicagotribune.com/business/ct-prem-biz-cta-bus-l-capacity-limit-20210611-evl5yvjxi5e3fobsy7qklto4ca-story.html>.

14. Coronavirus: Operations: Chicago Transit Authority; [cited 2023 April 11]. Available from: <https://www.transitchicago.com/coronavirus/operations/#:~:text=Effective%20June%2011%2C%20with%20the,to%20run%20at%20full%20capacity>.

15. New CTA rail cars make their debut 2010 [updated April 19; cited 2023 April 25]. Available from: <https://web.archive.org/web/20100422211206/http://www.chicagobreakingnews.com/2010/04/new-cta-rail-cars-make-their-debut.html>.

16. Klaassen F, Chitwood MH, Cohen T, Pitzer VE, Russi M, Swartwood NA, et al. Population immunity to pre-Omicron and Omicron SARS-CoV-2 variants in US states and counties through December 1, 2021. Clin Infect Dis. 2022. Epub 20220620. doi: 10.1093/cid/ciac438. PubMed PMID: 35717642; PubMed Central PMCID: PMCPMC9214178.

17. Klaassen F, Chitwood MH, Cohen T, Pitzer VE, Russi M, Swartwood NA, et al. Changes in Population Immunity Against Infection and Severe Disease From Severe Acute Respiratory Syndrome Coronavirus 2 Omicron Variants in the United States Between December 2021 and November 2022. Clin Infect Dis. 2023;77(3):355-61. doi: 10.1093/cid/ciad210. PubMed PMID: 37074868; PubMed Central PMCID: PMCPMC10425195.

18. Campbell F, Archer B, Laurenson-Schafer H, Jinnai Y, Konings F, Batra N, et al. Increased transmissibility and global spread of SARS-CoV-2 variants of concern as at June 2021. Euro Surveill. 2021;26(24). doi: 10.2807/1560-7917.Es.2021.26.24.2100509. PubMed PMID: 34142653; PubMed Central PMCID: PMCPMC8212592.

19. Earnest R, Uddin R, Matluk N, Renzette N, Turbett SE, Siddle KJ, et al. Comparative transmissibility of SARS-CoV-2 variants Delta and Alpha in New England, USA. Cell Rep Med. 2022;3(4):100583. Epub 20220311. doi: 10.1016/j.xcrm.2022.100583. PubMed PMID: 35480627; PubMed Central PMCID: PMCPMC8913280.

20. Burki TK. Omicron variant and booster COVID-19 vaccines. Lancet Respir Med. 2022;10(2):e17. Epub 20211217. doi: 10.1016/s2213-2600(21)00559-2. PubMed PMID: 34929158; PubMed Central PMCID: PMCPMC8683118.

21. Davies A, Thompson KA, Giri K, Kafatos G, Walker J, Bennett A. Testing the efficacy of homemade masks: would they protect in an influenza pandemic? Disaster Med Public Health Prep. 2013;7(4):413-8. doi: 10.1017/dmp.2013.43. PubMed PMID: 24229526; PubMed Central PMCID: PMCPMC7108646.

22. Milton DK, Fabian MP, Cowling BJ, Grantham ML, McDevitt JJ. Influenza virus aerosols in human exhaled breath: particle size, culturability, and effect of surgical masks. PLoS Pathog. 2013;9(3):e1003205. Epub 20130307. doi: 10.1371/journal.ppat.1003205. PubMed PMID: 23505369; PubMed Central PMCID: PMCPMC3591312.

23. Fischer CB, Adrien N, Silguero JJ, Hopper JJ, Chowdhury AI, Werler MM. Mask adherence and rate of COVID-19 across the United States. PLoS One. 2021;16(4):e0249891. Epub 20210414. doi: 10.1371/journal.pone.0249891. PubMed PMID: 33852626; PubMed Central PMCID: PMCPMC8046247.

24. State Population Totals and Components of Change: 2020-2022: United States Census Bureau; 2023 [updated March 23; cited 2023 April 7]. Available from: <https://www.census.gov/data/tables/time-series/demo/popest/2020s-state-total.html>.

25. COVID-19 Projections: Mask use: Institute for Health Metrics and Evaluation (IHME); [cited 2022 Nov 14]. Available from: <https://covid19.healthdata.org/united-states-of-america?view=mask-use&tab=trend>.

26. Team C-F. Variation in the COVID-19 infection-fatality ratio by age, time, and geography during the pre-vaccine era: a systematic analysis. Lancet. 2022;399(10334):1469-88. Epub 20220224. doi: 10.1016/S0140-6736(21)02867-1. PubMed PMID: 35219376; PubMed Central PMCID: PMCPMC8871594.

27. Sullivan PS, Siegler AJ, Shioda K, Hall EW, Bradley H, Sanchez T, et al. Severe Acute Respiratory Syndrome Coronavirus 2 Cumulative Incidence, United States, August 2020-December 2020. Clin Infect Dis. 2022;74(7):1141-50. doi: 10.1093/cid/ciab626. PubMed PMID: 34245245; PubMed Central PMCID: PMCPMC8406864.

28. Sigal A, Milo R, Jassat W. Estimating disease severity of Omicron and Delta SARS-CoV-2 infections. Nat Rev Immunol. 2022;22(5):267-9. doi: 10.1038/s41577-022-00720-5. PubMed PMID: 35414124; PubMed Central PMCID: PMCPMC9002222.

29. COVID-19 Results Briefing: IHME; [cited 2023 December 2, 2022]. Available from: <https://www.healthdata.org/sites/default/files/files/1_briefing_Global_6.pdf>.

30. What Is the Death Rate of Omicron? It’s WAY Less Than You Think [updated January 30, 2022April 6, 2023]. Available from: <https://centerforneurologyandspine.com/what-is-the-death-rate-of-omicron-its-way-less-than-you-think/>.

31. COVID Data Tracker: Trends in Number of COVID-19 Cases and Deaths in the US Reported to CDC: Centers for Disease Control and Prevention; [January 27, 2023]. Available from: <https://covid.cdc.gov/covid-data-tracker/#trends_weeklydeaths_newhospitaladmissions_00>.

32. Peng Z, Jimenez JL. Exhaled CO2 as a COVID-19 Infection Risk Proxy for Different Indoor Environments and Activities. Environmental Science & Technology Letters. 2021;8(5):392-7. doi: 10.1021/acs.estlett.1c00183.

33. The National Transit Database (NTD): Monthly Ridership: Federal Transit Administration; [October 28, 2022]. Available from: <https://www.transit.dot.gov/ntd/ntd-data?field_data_categories_target_id%5B2536%5D=2536&field_product_type_target_id=All&year=all&combine>=.

34. A C Line Review: NYC Transit Loading Guidlines: MTA New York City Transit; 2016 [updated February 23; cited 2023 May 18]. Available from: <http://web.mta.info/nyct/service/A-C_Pres_2016-02-23_rev.pdf>.

35. Klaassen F, Chitwood MH, Cohen T, Pitzer VE, Russi M, Swartwood NA, et al. Changes in population immunity against infection and severe disease from SARS-CoV-2 Omicron variants in the United States between December 2021 and November 2022. medRxiv. 2022. Epub 20221123. doi: 10.1101/2022.11.19.22282525. PubMed PMID: 36451882; PubMed Central PMCID: PMCPMC9709792.

36. SARS-CoV-2 Variant Classifications and Definitions: Centers for Disease Control and Prevention; [updated March 20, 2023May 5, 2023]. Available from: <https://www.cdc.gov/coronavirus/2019-ncov/variants/variant-classifications.html#anchor_1679059484954>.

37. Lau H, Khosrawipour T, Kocbach P, Ichii H, Bania J, Khosrawipour V. Evaluating the massive underreporting and undertesting of COVID-19 cases in multiple global epicenters. Pulmonology. 2021;27(2):110-5. Epub 20200606. doi: 10.1016/j.pulmoe.2020.05.015. PubMed PMID: 32540223; PubMed Central PMCID: PMCPMC7275155.

38. Fisher D, Wilder-Smith A. The global community needs to swiftly ramp up the response to contain COVID-19. Lancet. 2020;395(10230):1109-10. Epub 20200319. doi: 10.1016/S0140-6736(20)30679-6. PubMed PMID: 32199470; PubMed Central PMCID: PMCPMC7138255.

39. Ending Isolation and Precautions for People with COVID-19: Interim Guidance: Centers for Disease Control and Prevention; 2022 [updated Aug 31, 2022April 6, 2023]. Available from: <https://www.cdc.gov/coronavirus/2019-ncov/hcp/duration-isolation.html#:~:text=People%20with%20moderate%20or%20severe,for%20up%20to%2020%20days>.

40. Amazon marketplace [cited 2023 March 21]. Available from: <https://www.amazon.com/s?k=face+mask&crid=2F5O5DE1DV7BG&sprefix=face+mask%2Caps%2C104&ref=nb_sb_noss_1>.

41. Rice KL, Miller GF, Coronado F, Meltzer MI. Estimated Resource Costs for Implementation of CDC's Recommended COVID-19 Mitigation Strategies in Pre-Kindergarten through Grade 12 Public Schools - United States, 2020-21 School Year. MMWR Morb Mortal Wkly Rep. 2020;69(50):1917-21. Epub 20201218. doi: 10.15585/mmwr.mm6950e1. PubMed PMID: 33332295; PubMed Central PMCID: PMCPMC7745954.

42. Spennemann DHR. Facing COVID-19: Quantifying the Use of Reusable vs. Disposable Facemasks. Hygiene. 2021;1(3):120-8. doi: <https://doi.org/10.3390/hygiene1030011>.

43. Pan JY, Liu D. Mask-wearing intentions on airplanes during COVID-19 - Application of theory of planned behavior model. Transp Policy (Oxf). 2022;119:32-44. Epub 20220214. doi: 10.1016/j.tranpol.2022.01.023. PubMed PMID: 35185300; PubMed Central PMCID: PMCPMC8841390.

44. Everything you need to know about fares and tolls in New York: Metropolitan Transportation Authority (MTA); [cited 2023 April 3]. Available from: <https://new.mta.info/fares>.

45. Cost to Ride: Washington Metropolitan Area Transit Authority (WMATA); [cited 2023 April 3]. Available from: <https://www.wmata.com/fares/basic.cfm>.

46. Poster Prints: FedEx Office; [cited 2023 March 31]. Available from: <https://www.office.fedex.com/default/posters.html>.

47. New York City Transit: Metropolitan Transportation Authority (MTA). Available from: <https://new.mta.info/agency/new-york-city-transit>.

48. Facts at a glance: Chicago Transit Authority (CTA); [cited 2023 March 31]. Available from: <https://www.transitchicago.com/facts/>.

49. Railcar and Railcar Facilities Investments: Washington Metropolitan Area Transit Authority; [cited 2023 March 31]. Available from: <https://www.wmata.com/initiatives/capital-improvement-program/investments/Railcar-Investments.cfm#:~:text=Metro%20operates%20over%201%2C200%20railcars,in%20railcars%20and%20associated%20facilities>.

50. COVID-19 Cost Tracker: COVID-19 Medical and Hospitalization Costs: National: FAIR Health; 2021 [cited 2023 Mar 13,]. Available from: <https://s3.amazonaws.com/media2.fairhealth.org/infographic/asset/COVID-19%20Medical%20Hospitalization%20Costs%20by%20State%20-%20FINAL_National.pdf>.

51. Weiner JP, Bandeian S, Hatef E, Lans D, Liu A, Lemke KW. In-Person and Telehealth Ambulatory Contacts and Costs in a Large US Insured Cohort Before and During the COVID-19 Pandemic. JAMA Netw Open. 2021;4(3):e212618. Epub 20210301. doi: 10.1001/jamanetworkopen.2021.2618. PubMed PMID: 33755167; PubMed Central PMCID: PMCPMC7988360.

52. Bartsch SM, Ferguson MC, McKinnell JA, O'Shea KJ, Wedlock PT, Siegmund SS, Lee BY. The Potential Health Care Costs And Resource Use Associated With COVID-19 In The United States. Health Aff (Millwood). 2020;39(6):927-35. Epub 20200423. doi: 10.1377/hlthaff.2020.00426. PubMed PMID: 32324428.

53. Shrestha SS, Kompaniyets L, Grosse SD, Harris AM, Baggs J, Sircar K, Gundlapalli AV. Estimation of Coronavirus Disease 2019 Hospitalization Costs From a Large Electronic Administrative Discharge Database, March 2020-July 2021. Open Forum Infect Dis. 2021;8(12):ofab561. Epub 20211220. doi: 10.1093/ofid/ofab561. PubMed PMID: 34938822; PubMed Central PMCID: PMCPMC8686820.

54. Ohsfeldt RL, Choong CK, Mc Collam PL, Abedtash H, Kelton KA, Burge R. Inpatient Hospital Costs for COVID-19 Patients in the United States. Adv Ther. 2021;38(11):5557-95. Epub 20211005. doi: 10.1007/s12325-021-01887-4. PubMed PMID: 34609704; PubMed Central PMCID: PMCPMC8491188.

55. Wager E, Claxton G, Amin K, Cox C. Cost of COVID-19 hospital admissions among people with private health coverage: The Peterson Center on Healthcare and Kaiser Family Foundation; 2022 [Mar 13, 2023]. Available from: <https://www.healthsystemtracker.org/brief/cost-of-covid-19-hospital-admissions-among-people-with-private-health-coverage/#Average%20costs%20for%20inpatient%20COVID-19%20admissions%20among%20people%20with%20large%20employer%20health%20coverage,%202020%C2%A0>.

56. Tsai Y, Vogt TM, Zhou F. Patient Characteristics and Costs Associated With COVID-19-Related Medical Care Among Medicare Fee-for-Service Beneficiaries. Ann Intern Med. 2021;174(8):1101-9. Epub 20210601. doi: 10.7326/m21-1102. PubMed PMID: 34058109; PubMed Central PMCID: PMCPMC8252832.

57. NCHS Urban-Rural Classification Scheme for Counties Hyattsville, MD: National Center for Health Statistics; 2017 [cited 2023 November 8]. Available from: <https://www.cdc.gov/nchs/data_access/urban_rural.htm>.

58. Anzalone AJ, Horswell R, Hendricks BM, Chu S, Hillegass WB, Beasley WH, et al. Higher hospitalization and mortality rates among SARS-CoV-2-infected persons in rural America. J Rural Health. 2023;39(1):39-54. Epub 20220627. doi: 10.1111/jrh.12689. PubMed PMID: 35758856; PubMed Central PMCID: PMCPMC9349606.

59. County Population Totals and Components of Change: 2020-2022 2023 [updated June 13; cited 2023 November 8]. Available from: <https://www.census.gov/data/tables/time-series/demo/popest/2020s-counties-total.html>.

60. Weekly United States COVID-19 Cases and Deaths by County - ARCHIVED: Centers for Disease Controal and Prevention; 2023 [updated September 8; cited 2023 November 8]. Available from: <https://data.cdc.gov/dataset/Weekly-United-States-COVID-19-Cases-and-Deaths-by-/yviw-z6j5>.

61. COVID-19 Reported Patient Impact and Hospital Capacity by Facility: U.S. Department of Health & Human Services; 2023 [updated November 3; cited 2023 November 9]. Available from: <https://healthdata.gov/Hospital/COVID-19-Reported-Patient-Impact-and-Hospital-Capa/anag-cw7u>.
